# Association between thyroid hormone and cardiovascular health: A cross-sectional study

**DOI:** 10.1371/journal.pone.0329194

**Published:** 2025-10-24

**Authors:** Tongtong Bai, Juanjuan Peng, Chengyu Wu, Tonghua Liu

**Affiliations:** 1 Dongfang Hospital, Beijing University of Chinese Medicine, Beijing, China; 2 School of Acupuncture-Moxibustion and Tuina & School of Regimen and Rehabilitation, Nanjing University of Chinese Medicine, Nanjing, Jiangsu, China; 3 School of Chinese Medicine, Nanjing University of Chinese Medicine, Nanjing, Jiangsu, China; 4 Key Laboratory of Health Cultivation of the Ministry of Education, Beijing University of Chinese Medicine, Beijing, China; NUST: National University of Sciences and Technology, PAKISTAN

## Abstract

**Background:**

Large-sample clinical research evidence in humans regarding the association between thyroid hormones and cardiovascular health is limited. The American Heart Association introduced the Life’s Essential 8 (LE8) as a novel metric for assessing cardiovascular health. This study aimed to explore the plausible connection between thyroid hormone levels and LE8.

**Methods:**

This study employed data extracted from the National Health and Nutrition Examination Survey spanning from 2007–2012, focusing on individuals aged 20 and above. To investigate the association between thyroid hormone and LE8, diverse analytical methods, including weighted multivariate linear regression, restricted cubic spline curves, and stratified analysis, were utilized.

**Results:**

A total of 3,019 participants were enrolled in this study. The highest LE8 score group (≥ 80) comprised 610 participants (20.21%). In the fully adjusted linear regression analysis, elevated levels of ln(FT3), ln(TT3), and ln(Tg) were significantly associated with a reduced level of LE8 (*β* (95% Confidence Interval): −6.31 (−12.13, −0.49), p = 0.035; −5.18 (−8.22, −2.15), p = 0.002; −0.98 (−1.74, −0.22), p = 0.014). The analysis revealed nonlinear relationships between ln(Tg), ln(TgAb), ln(TPOAb), and LE8. In the group with normal thyroid hormone levels, ln(TT3), ln(TT4), and ln(Tg) correlated with LE8 in the stratified analysis.

**Conclusion:**

Alterations in FT3, TT3, TT4, Tg, TgAb, and TPOAb levels correlated with variations in LE8. Adherence to cardiovascular health recommendations may be pertinent to the preservation of thyroid health.

## Introduction

Thyroid hormone (TH) is an extracellular lipophilic signaling molecule that exerts a pervasive influence on cellular functionality in the human body. Its synthesis is meticulously controlled through the hypothalamic-pituitary-thyroid axis [1,2]. The sequence is initiated within the hypothalamus where thyrotropin-releasing hormone (TRH) synthesis commences [2], stimulating the anterior pituitary gland to release thyroid-stimulating hormone (TSH) [3]. Following its release, the thyroid gland autonomously synthesizes and secretes triiodothyronine (T3) and thyroxine (T4) [4]. Elevated circulating levels of TH suppress the secretion of TRH from the hypothalamus and TSH from the pituitary gland, establishing a negative feedback loop that regulates their production [4,5]. T4, the predominant secretory output of the thyroid, exhibits relatively low activity and is converted to the active hormone T3 [6]. Although a portion of T3 originates within the thyroid, approximately 80% is synthesized in extrathyroidal tissues via the deiodination of T4 [2,5]. The majority of both T4 and T3 produced binds to thyroxine-binding globulin and other proteins, representing a reservoir of stored hormones that are biologically inactive [7,8]. The regulatory mechanisms of the hypothalamic-pituitary-thyroid axis and peripheral organ-level metabolism of THs are adaptable and dynamic, potentially optimizing the organism’s response to various changes and challenges [9]. Dynamic alterations in TH are closely linked to cardiovascular physiology and pathophysiology [10].

Cardiovascular disease (CVD) is the leading cause of mortality worldwide, and its incidence and associated fatalities impose a significant burden [11]. Previous research has identified free thyroxine (FT4) as an independent predictor of cardiovascular mortality and risk of CVD [12]. The correlation between FT4 levels and CVD showed a J-shaped pattern [13]. Another TH, TSH, was also associated with CVD, with low TSH concentrations demonstrating an increased risk of cardiovascular mortality [13]. Adherent healthy behaviors and salutogenic factors are correlated with cardiovascular health and contribute significantly to the prevention of CVD [14]. In 2022, the American Heart Association (AHA) introduced Life’s Essential 8 (LE8) health metrics to enhance cardiovascular health assessment, building upon the Life’s Simple 7 (LS7) framework established in 2010 [15]. LE8 incorporated sleep quality as a metric and refined the scoring algorithm to provide a more comprehensive evaluation of cardiovascular health [15]. However, research on the correlation between TH and LE8 in the general population is scarce.

This study aimed to elucidate the plausible associations between TH and LE8 in the United States (U.S.). We hypothesized that a correlation between TH and LE8 levels would be detected, suggesting that cardiovascular health is associated with TH.

## Materials and methods

### Data sources and study population

The dataset used in this investigation was exclusively sourced from the National Health and Nutrition Examination Survey (NHANES) website [16]. The NHANES orchestrated by The National Center for Health Statistics (NCHS) is an all-encompassing national survey specifically crafted to obtain data emblematic of the population. This study utilized a sophisticated stratified multistage probabilistic cluster sampling technique to conduct an exhaustive evaluation of the health and nutritional status of the general population throughout the U.S. [17]. In this cross-sectional investigation, we utilized an aggregated 6-year dataset for our analyses to ensure robust and accurate estimates. Data acquisition was conducted in early May 2024. Data analysis was conducted in May 2024. Before participating in the study, all individuals provided written informed consent, and the NHANES investigation obtained approval from the Ethics Review Board of NCHS [18]. During or after data collection, the authors did not have access to information that could identify individual participants. The NHANES study was authorized by the NCHS Ethics Review Committee, and all participants provided written informed consent prior to their participation. This study was exempted from ethical review and approval for secondary analysis, as it was determined that no supplementary institutional review board clearance was required. This study was conducted in accordance with the guidelines of the Declaration of Helsinki. We employed a weighted analysis to ensure that the conclusions drawn from our sample-based analysis were generalizable to the overall U.S. population.

**Fig 1:**
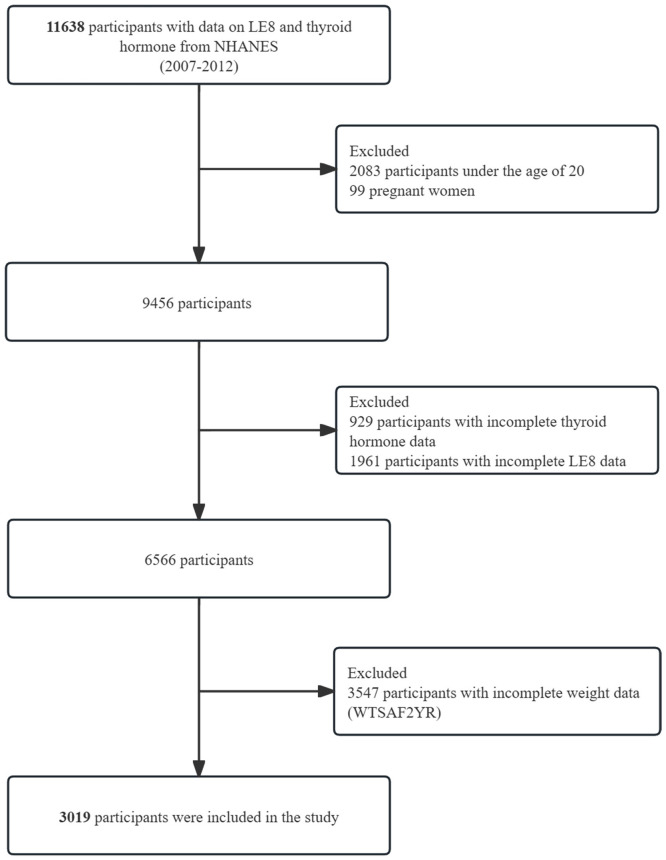
Flow chart of patient selection.

Our analysis used data collected from participants spanning three biennial NHANES cycles (2007–2008, 2009–2010, and 2011–2012). Within the NHANES 2007–2012 dataset, 11,638 participants met the inclusion criteria and possessed complete data on TH measurements and LE8 metrics. Participants were excluded if they fell under the following categories: under the age of 20 years, pregnant, had missing data for the TH or LE8 tests, or had incomplete weight information. Ultimately, the analysis encompassed a total of 3,019 participants within the age of 20 years old and above. A visual representation outlining the selection methodology employed for the study participants is presented in **Fig 1**.

### Clarification of the definitions of thyroid hormones and Life’s Essential 8

The AHA initially defined the cardiovascular health paradigm in 2010 based on seven health behaviors and factors (collectively termed LS7) associated with greater CVD-free survival, total lifespan, and quality of life [14]. In 2022, the AHA’s LE8 initiative was a critical advancement in the enhancement of cardiovascular health with components including diet, physical activity, nicotine exposure, sleep health, body mass index (BMI), blood lipids, blood glucose, and blood pressure [15]. Compared to the LS7, LE8 incorporates an additional metric of sleep health, building upon the original cardiovascular health framework [15]. LE8, an enhanced and comprehensive set of health metrics and assessment frameworks, has been used extensively in population-based studies [19–21]. This study employed meticulous data collection techniques as previously documented in the literature. These techniques included the use of dietary questionnaires to determine the Dietary Approaches to Stop Hypertension (DASH) diet score; self-reported minutes per week of moderate to vigorous physical activity; exposure to tobacco and nicotine; length of sleep; and measurements of blood pressure, weight, height, blood glucose, and non-high-density lipoprotein cholesterol (non-HDL) levels [15]. Each metric was evaluated using a scoring algorithm and the aggregate cardiovascular health score was derived from the unweighted mean of these metrics, with scores ranging from 0 to 100 points. Based on literature findings, the LE8 score was categorized into low (< 50), moderate (50–80), and high groups (≥ 80) [15,22]. The LE8 represents a comprehensive assessment of cardiovascular health, integrating both self-reported and objective measures to provide a critical evaluation of health factors essential for cardiovascular risk [15,23].

Serum TH levels were used to evaluate thyroid function. The TH profile encompasses a series of tests aimed at assessing thyroid function, including levels of total thyroxine (TT4), FT4, total triiodothyronine (TT3), FT3, thyroglobulin (TG), thyroglobulin antibodies (TgAbs), thyroid peroxidase antibodies (TPOAb), and TSH. To establish population-based reference ranges for these hormone levels, comprehensive instructions regarding specimen collection and processing were derived from the Laboratory Methods Manual and Laboratory/Medical Technologists Procedure Manual available on the NHANES website [24]. Specifically, the reference ranges for TH levels are as follows: TSH levels (measured in uIU/mL) between 0.24–5.4 define the normal level group, below 0.24 define the low-level group, and above 5.4 define the high-level group. FT3 levels (measured in pg/mL) between 2.5–3.9 define the normal level group, below 2.5 the low-level group, and above 3.9 the high-level group. FT4 levels (measured in ng/dL) are considered normal between 0.6–1.6, below 0.6 indicate a lower level, and above 1.6 indicate a higher level. TT3 levels (measured in ng/dL) fall within the normal range at 87–178, below 87 ng/dL suggest a lower level, and above 178 ng/dL suggest a higher level. TT4 levels (measured in ug/dL) are within the normal range at 6.1–12.2, below 6.1 indicate a lower level, and above 12.2 indicate a higher level. TG levels (measured in ng/mL) are considered normal below 35.0 and categorized as high at or above 35.0. TgAb levels (measured in IU/mL) are within the normal range at < 4.0 and are classified as high at or above 4.0. TPOAb levels (measured in IU/mL) are considered normal below 9.0 and categorized as high at or above 9.0.

### Covariates

Covariates were selected based on three criteria: (1) inclusion of established confounders informed by prior evidence of thyroid-cardiovascular associations, clinical relevance, and sufficient outcome events; (2) variables altering effect estimates by > 10% when added to or removed from core models; and (3) variables with p < 0.1 in univariate screening. Multivariate linear regression models were adjusted for demographic characteristics, clinical biomarkers, self-reported comorbidities, and medication use. A multitude of potential confounding variables was scrutinized, drawing from the extant literature on TH or LE8 [12,25–31]. A comprehensive range of covariates was considered, encompassing demographic factors such as age (in years), sex (male and female), race/ethnicity (categorized as non-Hispanic White, non-Hispanic Black, Mexican American, other Hispanic, other race-including Multi-Racial), educational attainment (three segments: less than 9th grade, 9th grade-high school grade, or more than high school grade), marital status (four categories: married, never married, living with partner, or other including widowed, divorced, or separated individuals), poverty income ratio (PIR) divided into low (≤ 1.30), medium (1.31–3.50), and high (> 3.50), and alcohol user (never: had < 12 drinks in lifetime; former: had ≥ 12 drinks in 1 year and did not drink last year, or did not drink last year but drank ≥ 12 drinks in lifetime; current heavy alcohol user: ≥ 3 drinks per day for females, ≥ 4 drinks per day for males, or binge drinking ≥ 4 drinks on same occasion for females or ≥ 5 drinks on same occasion for males on 5 or more days per month; current moderate alcohol use: ≥ 2 drinks per day for females, ≥ 3 drinks per day for males, or binge drinking ≥ 2 days per month; current mild alcohol use: ≤ 1 drinks per day for females, ≤ 2 drinks per day for males) [30,32]. Biomedical indicators were also noted including uric acid (mg/dL), creatinine (mg/dL), alanine aminotransferase (Alt, [U/L]), aspartate aminotransferase (Ast, [U/L]), and urine Iodine (ug/L) [30]. Thyroid disease status was determined using two validated questions: “Do you currently have any thyroid problems?” or “Have you ever been diagnosed with thyroid cancer?” [33]. If the answer to either of the two questions was “Yes,” this variable was classified as “having thyroid disease”; otherwise, it was defined as “No.” CVD was defined as a self-reported condition diagnosed by a physician, including congestive heart failure, coronary heart disease, angina, heart attack, and stroke [34]. Medications associated with thyroid dysfunction, TH modulation, and thyroid disease were identified using these two approaches (see S1 Table). First, we referenced prescription medication records corresponding to thyroid diseases from the International Classification of Diseases-Tenth Revision-Clinical Modification (ICD-10-CM) information in other years of the NHANES database [35,36]. Second, we consulted previously published literature that mentioned the names of medications related to THs in the NHANES dataset [31,37–41].

### Statistical analysis

This study re-examined datasets that were openly accessible for analysis purposes by employing a weighted analysis. Due to the absence of weights for thyroid testing included in the 2007–2008 cycle (WTSA2YR) and the inclusion of fasting blood glucose data in LE8, the analysis was conducted using the Fasting Subsample 2 Year Mobile Examination Center (MEC) Weight as the analytical weight (WTSAF2YR). The weights were calculated by dividing the WTSAF2YR values by the number of 2-year cycles included in the analysis (WTSAF2YR/3). Normality assessment for continuous variables was conducted using two methods: visualizing the variables through histograms and examining whether the mean exceeded twice the standard deviation (see S1 Fig). Participant characteristics are presented as means with standard deviations (for normally distributed continuous variables) or medians with interquartile ranges (for non-normally distributed continuous variables). Categorical variables are represented as counts and percentages, as appropriate. LE8 was examined in both continuous and categorical forms. THs were processed for analysis as follows: they were transformed using logarithmic conversion, treated as continuous variables, and categorized into subgroups based on hormone reference ranges, specifically the normal range, low-level group, and high-level group.

To evaluate the differences among groups, we employed various statistical techniques: weighted one-way analysis of variance (suitable for normally distributed data), weighted Kruskal-Wallis tests (applied to non-normally distributed data), and weighted chi-square tests (used for categorical variables). Post hoc analysis was conducted using Tukey’s honest significant difference (HSD) method for pairwise comparisons, and the results were visualized using box plots to illustrate intergroup differences (see S2–S7 Figs). Additionally, we employed weighted linear regression models to determine effect sizes (*β*) and 95 percent confidence intervals (95% CI) while examining the association between TH (after log transformation) and LE8, as well as the association between LE8 and covariates. Before constructing the multivariate model, we assessed the collinearity between LE8 and other variables by calculating the generalized variance inflation factor (GVIF) (see S2 Table). A variable was considered significantly collinear if the GVIF^(1/2Df) was equal to or greater than two degrees of freedom (Df). We used three models to adjust for various covariates, thereby elucidating the interrelationship between variables and LE8. In Model 1, statistical adjustments were applied specifically to sociodemographic factors, including age, sex, and race/ethnicity. Model 2 expanded these adjustments to include age, sex, race/ethnicity, and urinary iodine. Model 3 (fully adjusted model) was adjusted for age, sex, race/ethnicity, urine iodine, education, marital status, PIR, CVD, creatinine, uric acid, Alt, AST, alcohol use, prescription drugs affecting thyroid function, and thyroid disease. A residual diagnosis was made for the fully adjusted linear regression model to validate its robustness by assessing whether the model residuals followed a normal distribution (see S8–S15 Figs). For all primary analyses, we used the E-value to assess the validity against unmeasured confounders [42,43]. The E-value can test the strength of the association between exposure and outcome while evaluating the evidence of causality. Unmeasured confounders must be associated with both THs and LE8, with a risk ratio equal to the E-value, to fully explain the observed association between THs and cardiovascular health. Additionally, the association between serum TH levels (after log transformation) and LE8 score was examined for potential nonlinearity. Restrictive cubic spline (RCS) analysis was used to delineate the nonlinear relationships between these two variables. The capping method was utilized by applying a 2.5% tail trimming to address extreme values in the RCS analysis.

In the sensitivity analysis, we examined the relationship between a specific TH (after log transformation) and LE8 after grouping according to the reference range of TH. If the sample size within a subgroup was insufficient, the analysis was omitted. To validate the robustness of the results, the sensitivity analysis also included a fully adjusted linear regression analysis, excluding participants with a history of CVD and thyroid disease. Single imputation methods were employed to handle missing data. Data processed using various missing value handling methods and different weighting analyses were employed for the sensitivity analyses in the fully adjusted model. All reported probabilities (p values) were two-sided, and statistical significance was established at a threshold of p < 0.05. We performed analyses using R software (version available at http://www.R-project.org, provided by The R Foundation) and Free Statistics software version 1.9.2. During the preparation for this study, the authors used Kimi (Moonshot AI) and DeepSeek (AI) to improve language and readability.

## Results

### Study population

Of the 11,638 participants who underwent interviews, 2,083 were less than 20-years-old. Exclusions were made for pregnant women (n = 99), individuals with missing data for TH (n = 929) and incomplete LE8 data (n = 1,961), as well as those with incomplete weight information (n = 3,547). Consequently, the analysis encompassed 3,019 participants from the NHANES dataset from the 2007–2012. study. **Fig 1** shows a detailed and fully comprehensive representation of the inclusion and exclusion criteria.

### Baseline characteristics

**Table 1** presents an exhaustive overview with the results of the weighted analysis elucidating the foundational characteristics of the entire subject pool segregated by their LE8 scores (the demarcation points situated at 50 and 80). The results indicate a total participant pool of 3,019 individuals, with 386 in the low category (< 50), 2,023 in the moderate group (50–80), and 610 in the high category (≥ 80). The mean age of the participants was 47.12 (± 16.44) years and 1,530 individuals (51.4%) identified as women. Individuals with a high LE8 score were more likely to be younger, female, of Non-Hispanic White background, have an educational attainment exceeding 9th grade, be married, have a high income, be mild alcohol users, and not have CVD. Additionally, they exhibited lower levels of Alt, Ast, uric acid, creatinine, and urine iodine; did not use prescription medications; and had no thyroid diseases. The results of the post-hoc test provided a clear illustration of the pairwise comparisons between the groups (see S2–S7 Figs). Visualization of the eight TH graphs indicated that TSH, Tg, TGAb, and TPOAb followed a non-normal distribution (see S1 Fig). Therefore, to facilitate the analysis, a logarithmic transformation using the natural base (e) was applied to all TH variables.

**Table 1:** Demographic profiles and initial participant attributes in the current research inquiry.

Characteristic	LE8 total score				p-Value
	Total	<50	50-80	≥80	
**NO.**	n = 3019	n = 386	n = 2023	n = 610	
**Age** (years), Mean±SD	47.51 ± 16.69	55.42 ± 14.50	48.40 ± 16.53	41.75 ± 16.15	<0.001
**Sex**, n (%)					<0.001
Men	1489 (48.6)	186 (46.1)	1058 (52.8)	245 (38.5)	
Women	1530 (51.4)	200 (53.9)	965 (47.2)	365 (61.5)	
**Race/ethnicity,** n (%)					0.003
Non-Hispanic White	1502 (72.4)	189 (70.7)	1004 (72.1)	309 (73.9)	
Non-Hispanic Black	542 (10.1)	94 (14.1)	387 (11.3)	61 (5.2)	
Mexican American	464 (7.3)	44 (5.6)	315 (7.1)	105 (8.3)	
Other Hispanic	334 (4.8)	47 (5.6)	213 (4.6)	74 (5.1)	
Other Race – Including Multi-Racial	177 (5.4)	12 (3.9)	104 (4.9)	61 (7.5)	
**Education**, n (%)					<0.001
Less Than 9th Grade	805 (17.6)	164 (32.5)	543 (18.5)	98 (8.6)	
9th Grade – High School Grade	705 (22.8)	110 (32.2)	500 (24.5)	95 (14.1)	
More Than High School Grade	1509 (59.6)	112 (35.3)	980 (57)	417 (77.4)	
**Marital status**, n (%)					<0.001
Married	1609 (57.0)	165 (47.0)	1097 (57.6)	347 (59.7)	
Never married	482 (16.8)	56 (13.3)	294 (15.6)	132 (21.8)	
Living with partner	240 (8.1)	29 (6.5)	159 (8.2)	52 (8.4)	
Other: widowed, divorced, or separated individuals	688 (18.1)	136 (33.2)	473 (18.6)	79 (10.1)	
**Poverty income ratio**, n (%)					<0.001
Low income (≤1.30)	923 (20.5)	189 (36.6)	611 (20.6)	123 (13.2)	
Medium income (1.31–3.50)	1166 (36.0)	146 (39.6)	784 (36.5)	236 (33.1)	
High income (>3.50)	930 (43.5)	51 (23.8)	628 (42.9)	251 (53.7)	
**Alcohol user**, n (%)					<0.001
Never	404 (10.6)	52 (11.9)	266 (10.4)	86 (10.7)	
Former	576 (16.1)	113 (27.5)	399 (17)	64 (8.7)	
Mild	1000 (35.9)	96 (25.7)	656 (34.3)	248 (44.8)	
Moderate	436 (16.8)	47 (13.9)	279 (16.2)	110 (19.6)	
Heavy	603 (20.6)	78 (21.1)	423 (22.2)	102 (16.1)	
**Uric acid** (mg/dl), Mean±SD	5.55 ± 1.39	6.03 ± 1.55	5.68 ± 1.36	4.97 ± 1.21	<0.001
**Creatinine** (mg/dl), Mean±SD	0.87 ± 0.29	0.91 ± 0.45	0.88 ± 0.28	0.82 ± 0.18	<0.001
**CVD**, n (%)					<0.001
No	2669 (91.1)	279 (75.0)	1806 (91.8)	584 (96.0)	
Yes	350 (8.9)	107 (25.0)	217 (8.2)	26 (4.0)	
**Alt** (U/L), Median (IQR)	21.00 (17.00, 29.00)	22.00 (17.00, 30.00)	22.00 (17.00, 30.00)	19.00 (16.00, 25.00)	<0.001
**Ast** (U/L), Median (IQR)	23.00 (20.00, 27.00)	23.00 (19.00, 27.00)	23.00 (20.00, 28.00)	22.00 (19.00, 26.00)	0.084
**Iodine, urine** (ug/L), Median (IQR)	141.71 (79.90,234.96)	158.46 (96.33,260.81)	143.80 (82.57,241.90)	128.77 (70.70,199.02)	0.016
**Prescription drugs affecting thyroid function**, n (%)					0.022
No	2764 (91.9)	336 (86.9)	1857 (92.1)	571 (93.6)	
Yes	255 (8.1)	50 (13.1)	166 (7.9)	39 (6.4)	
**Thyroid diseases**, n (%)					0.123
No	2712 (89.4)	341 (85.1)	1815 (89.7)	556 (90.4)	
Yes	307 (10.6)	45 (14.9)	208 (10.3)	54 (9.6)	
**TSH** (uIU/mL), Median (IQR)	1.69 (1.15,2.52)	1.75 (1.29,2.98)	1.70 (1.15,2.48)	1.58 (1.09,2.44)	0.170
**FT3** (pg/mL), Mean±SD	3.22 ± 0.50	3.18 ± 0.39	3.24 ± 0.56	3.19 ± 0.34	0.124
**FT4** (ng/dL), Mean±SD	0.80 ± 0.15	0.80 ± 0.15	0.80 ± 0.16	0.80 ± 0.13	0.817
**TT3** (ng/dL), Mean±SD	115.03 ± 23.60	113.78 ± 22.05	116.12 ± 24.36	112.57 ± 21.89	0.019
**TT4** (ug/dL), Mean±SD	7.89 ± 1.63	8.12 ± 1.71	7.89 ± 1.65	7.79 ± 1.53	0.075
**Tg** (ng/mL), Median (IQR)	9.98 (5.67,17.14)	13.17 (7.00,22.79)	10.09 (5.73,17.27)	9.16 (5.24,13.90)	<0.001
**TgAb** (IU/mL), Median (IQR)	0.60 (0.60,0.60)	0.60 (0.60,0.60)	0.60 (0.60,0.60)	0.60 (0.60,0.60)	0.236
**TPOAb** (IU/mL), Median (IQR)	0.70 (0.30,1.90)	0.60 (0.30,2.81)	0.60 (0.30,1.70)	0.70 (0.40,2.20)	0.046
**ln(TSH)**, Median (IQR)	0.52 (0.14,0.93)	0.56 (0.25,1.09)	0.53 (0.14,0.91)	0.46 (0.09,0.89)	0.170
**ln(FT3)**, Mean±SD	1.16 ± 0.12	1.15 ± 0.12	1.17 ± 0.13	1.15 ± 0.11	0.183
**ln(FT4)**, Median (IQR)	−0.22 (−0.36,-0.11)	−0.22 (−0.36,-0.11)	−0.22 (−0.36,-0.11)	−0.22 (−0.36,-0.11)	0.362
**ln(TT3)**, Mean±SD	4.73 ± 0.20	4.72 ± 0.19	4.73 ± 0.20	4.71 ± 0.18	0.031
**ln(TT4)**, Mean±SD	2.04 ± 0.20	2.07 ± 0.22	2.04 ± 0.21	2.03 ± 0.19	0.125
**ln(Tg)**, Median (IQR)	2.30 (1.73,2.84)	2.58 (1.95,3.13)	2.31 (1.75,2.85)	2.21 (1.66,2.63)	<0.001
**ln(TgAb)**, Median (IQR)	−0.51 (−0.51,-0.51)	−0.51 (−0.51,-0.51)	−0.51 (−0.51,-0.51)	−0.51 (−0.51,-0.51)	0.236
**ln(TPOAb)**, Median (IQR)	−0.36 (−1.20,0.64)	−0.51 (−1.20,1.03)	−0.51 (−1.20,0.53)	−0.36 (−0.92,0.79)	0.046

***Abbreviation:*** NO, number; LE8, life’s essential 8; SD, standard deviation; IQR, interquartile range; ALT, alanine aminotransferase; AST, aspartate aminotransferase; CVD, cardiovascular disease; TSH, thyroid-stimulating hormone; FT3, free triiodothyronine; FT4, free thyroxine; TT3, total triiodothyronine; TT4, total thyroxine; Tg, thyroglobulin; TgAb, thyroglobulin antibody; TPOAb, thyroid peroxidase antibody.

### Relationship between thyroid hormone and Life’s Essential 8

Weighted univariate analysis revealed associations between LE8 and several variables including age, sex, race/ethnicity, marital status, education level, PIR, alcohol use, Alt level, Ast level, uric acid level, creatinine level, urine iodine level, CVD, prescription drugs affecting thyroid function, and thyroid disease (see S3 Table).

The relationship between TH (after log transformation) and LE8 is presented in Table 2. In the non-adjusted model, ln(TT4) and ln(Tg) negatively correlated with LE8. However, ln(FT3) (*β* (95%CI): −6.31(−12.13, −0.49), p = 0.035), ln(TT3) (*β* (95%CI): −5.18(−8.22, −2.15), p = 0.002), and ln(Tg) (*β* (95%CI): −0.98(−1.74, −0.22), p = 0.014) were negatively correlated with LE8, after full adjustment for all covariates. The substantially elevated E-values suggest the robustness of these associations. The residuals of the linear regression model approximately followed a normal distribution (see S8–S15 Figs).

**Table 2:** Association between thyroid hormone and life’s essential 8 in different models.

Variables	LE8 total score											
	Non-adjusted Model			Model 1			Model 2			Model 3		
	*β* (95%CI)	p-Value	E-value (CI)	*β* (95%CI)	p-Value	E-value (CI)	*β* (95%CI)	p-Value	E-value (CI)	*β* (95%CI)	p-Value	E-value (CI)
**ln(TSH)**	−0.55(−1.60, 0.51)	0.305	/	−0.40(−1.38, 0.59)	0.421	/	−0.36(−1.39, 0.67)	0.482	/	−0.30(−1.15, 0.55)	0.474	/
**ln(FT3)**	−0.29(−5.48, 4.90)	0.911	/	−15.16(−21.68, −8.63)	<0.001	4.67 (2.85)	−15.46(−21.75, −9.17)	<0.001	4.77 (2.98)	−6.31(−12.13, −0.49)	0.035	2.35 (1.21)
**ln(FT4)**	−0.98(−4.69, 2.73)	0.598	/	0.34(−3.23, 3.91)	0.847	/	0.43(−3.07, 3.93)	0.804	/	1.90(−0.68, 4.48)	0.141	/
**ln(TT3)**	−1.65(−6.00, 2.70)	0.450	/	−8.48(−12.82, −4.15)	<0.001	2.82 (1.93)	−8.66(−12.79, −4.53)	<0.001	2.86 (2.00)	−5.18(−8.22, −2.15)	0.002	2.12 (1.55)
**ln(TT4)**	−4.94(−8.11, −1.76)	0.003	2.08 (1.48)	−5.41(−8.27, −2.55)	<0.001	2.17 (1.63)	−5.36(−8.23, −2.48)	<0.001	2.16 (1.62)	−2.04(−4.78, 0.71)	0.138	/
**ln(Tg)**	−1.00(−1.71, −0.30)	0.006	1.33 (1.16)	−1.14(−1.85, −0.44)	0.002	1.36 (1.20)	−1.13(−1.85, −0.42)	0.003	1.36 (1.19)	−0.98(−1.74, −0.22)	0.014	1.33 (1.13)
**ln(TgAb)**	0.18(−0.31, 0.67)	0.456	/	0.36(−0.06, 0.77)	0.090	/	0.36(−0.06, 0.78)	0.088	/	0.30(−0.17, 0.76)	0.196	/
**ln(TPOAb)**	0.24(−0.16, 0.64)	0.229	/	0.21(−0.14, 0.56)	0.229	/	0.21(−0.13, 0.56)	0.220	/	0.08(−0.26, 0.41)	0.651	/

***Abbreviation:*** CI, confidence interval; LE8, life’s essential 8; TSH, thyroid-stimulating hormone; FT3, free triiodothyronine; FT4, free thyroxine; TT3, total triiodothyronine; TT4, total thyroxine; Tg, thyroglobulin; TgAb, thyroglobulin antibody; TPOAb, thyroid peroxidase antibody.

The symbol “/” denotes statistically non-significant results, and calculation of the e-value is therefore omitted.

Model 1 was adjusted for age, gender, and race/ethnicity.

Model 2 was adjusted for age, sex, race/ethnicity, and urinary iodine levels.

Model 3 was adjusted for age, sex, race/ethnicity, urinary iodine level, education, marital status, PIR, CVD, creatinine, uric acid, Alt, Ast, alcohol use, prescription drugs affecting thyroid function, and thyroid disease.

**Fig 2:**
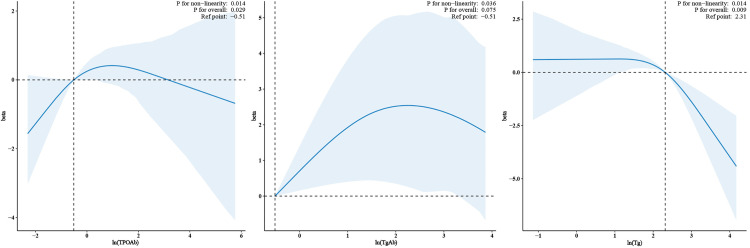
Restricted cubic spline plots between ln(Tg), ln(TgAb), ln(TPOAb), and LE8, with vertical lines indicating the cut points. ***Abbreviation:*** Tg, thyroglobulin; TgAb, thyroglobulin antibody; TPOAb, thyroid peroxidase antibody. The model was fully adjusted (corrected for age, sex, race/ethnicity, urine iodine, education, marital status, PIR, CVD, creatinine, uric acid, Alt, AST, alcohol use, prescription drugs affecting thyroid function, and thyroid disease). The extreme values (2.5% of each tail) were trimmed to preserve 95% of the participants.

To visualize the relationship between the TH levels (after log transformation) and LE8 scores, a nonlinearity test was conducted. For variables exhibiting a nonlinear relationship, the RCS was fitted as shown in **Fig 2**. Notably, nonlinear relationships were observed between ln(Tg), ln(TgAb), ln(TPOAb), and LE8. The relationships among ln(Tg), ln(TgAb), ln(TPOAb), and LE8 were approximately inverse L-shaped.

### Sensitivity analysis

To assess the robustness of the relationship between TH levels (after log transformation) and LE8 scores, subgroup analyses were performed to examine the association within the subgroups stratified by normal and abnormal hormone levels (**Table 3**). In the subgroup with normal TT3 levels, we found a significant negative correlation between ln(TT3) and LE8 (*β* (95% CI): −7.73 (−15.09, −0.37), p = 0.04). In the subgroup with normal TT4 levels, ln(TT4) showed a significant positive correlation with LE8 (*β* (95% CI): −7.59 (−14.32, −0.86), p = 0.03). Notably, in the subgroup with normal Tg levels, ln(Tg) exhibited a negative correlation with LE8 (*β* (95% CI): −1.53 (−2.85, −0.20), p = 0.03). The substantially elevated E-values suggest the strength of these associations. Linear regression analyses conducted after excluding populations with cardiovascular or thyroid diseases revealed a similar negative correlation, indicating the validity of the results (see S4 Table and S16–S17 Figs). Similarly, linear regression analyses using different missing data handling methods also demonstrated consistent negative correlations, further supporting the robustness of our findings (see S5 Table and S18–S20 Figs). Analyses incorporating MEC weights showed a comparable negative correlation between ln(FT3), ln(TT3), ln(Tg), and LE8 as well as an additional negative correlation between ln(TT4) and LE8. Moreover, ln(FT4) and ln(TPOAb) were positively correlated with LE8 levels. The explanation for this result may be that MEC weight analyses retained a larger sample size, with nearly double the number of participants in the SAF weight analyses.

**Table 3:** Association between different level groups of thyroid hormone and life’s essential 8.

Variables	Groups	NO.	LE8 total score			
			Adjusted Model			
			*β* (95%CI)	p-Value	E-value (CI)	*P for interaction*
**ln(TSH)**	**TSH** (uIU/mL)					0.061
	Normal (0.24–5.4)	2894	−0.90(−2.68, 0.88)	0.308	//	
	Low (<0.24)	40	/	/	//	
	High (>5.4)	85	/	/	//	
**ln(FT3)**	**FT3** (pg/mL)					0.027
	Normal (2.5–3.9)	2842	−7.64(−20.56, 5.28)	0.233	//	
	Low (<2.5)	54	/	/	//	
	High (>3.9)	123	/	/	//	
**ln(FT4)**	**FT4** (ng/dL)					0.911
	Normal (0.6–1.6)	2947	3.75(−1.35, 8.84)	0.142	//	
	Low (<0.6)	63	/	/	//	
	High (>1.6)	9	/	/	//	
**ln(TT3)**	**TT3** (ng/dL)					0.511
	Normal (87–178)	2736	−7.73(−15.09, −0.37)	0.040	2.65 (1.18)	
	Low (<87)	233	3.36(−22.38, 29.11)	0.735	//	
	High (>178)	50	/	/	//	
**ln(TT4)**	**TT4** (ug/dL)					0.353
	Normal (6.1–12.2)	2708	−7.59(−14.32, −0.86)	0.029	2.62 (1.30)	
	Low (<6.1)	260	13.48(−20.05, 47.02)	0.327	//	
	High (>12.2)	51	/	/	//	
**ln(Tg)**	**TG** (ng/mL)					0.465
	Normal (<35.0)	2792	−1.53(−2.85, −0.20)	0.026	1.44 (1.13)	
	High (≥35.0)	227	−6.38(−15.27, 2.51)	0.134	//	
**ln(TgAb)**	**TgAb** (IU/mL)					0.143
	Normal (<4.0)	2788	1.02(−2.11, 4.16)	0.506	//	
	High (≥4.0)	231	−1.57(−3.43, 0.28)	0.089	//	
**ln(TPOAb)**	**TPOAb** (IU/mL)					0.232
	Normal (<9.0)	2658	0.58(−0.29, 1.44)	0.184	//	
	High (≥9.0)	361	−1.05(−3.47, 1.37)	0.367	//	

***Abbreviation:*** NO, number; LE8, life’s essential 8; CI, confidence interval; TSH, thyroid-stimulating hormone; FT3, free triiodothyronine; FT4, free thyroxine; TT3, total triiodothyronine; TT4, total thyroxine; Tg, thyroglobulin; TgAb, thyroglobulin antibody; TPOAb, thyroid peroxidase antibody.

The symbol “/” indicates insufficient data, making it impossible to derive analytical results. The symbol “//” denotes statistically non-significant results, and calculation of the e-value is therefore omitted. The Model was adjusted for age, sex, race/ethnicity, urinary iodine level, education, marital status, PIR, CVD, creatinine, uric acid, Alt, Ast, alcohol use, prescription drugs affecting thyroid function, and thyroid diseases.

## Discussion

In this large U.S. population-based study, elevated FT3, TT3, and Tg levels were associated with reduced LE8 scores. ln(FT3), ln(TT3), and ln(Tg) were significantly negatively correlated with the total LE8 score. Notably, nonlinear relationships were observed between LE8 and ln(Tg), ln(TgAb), and ln(TPOAb). Furthermore, in the euthyroid reference range subgroup, pronounced negative correlations were observed between LE8 and ln(TT3), ln(TT4), and ln(Tg). Sensitivity analyses confirmed the robustness of the findings. To the best of our knowledge, this study is the first comprehensive investigation of the interrelationship between TH and LE8.

TH serve as pivotal regulators of cellular, tissue, and organ functions in the human body [44]. Receptors for TH are extensively distributed throughout the human body, with significant concentrations found in tissues such as the heart and vasculature [10]. The influence of THs on the cardiovascular system is intricate and multidimensional, with possible underlying pathways including endothelial impairment, modifications in arterial pressure, cardiac muscle dysfunction, and lipid metabolism disturbances [10]. Extensive prior research has consistently demonstrated a significant correlation between thyroid function and CVD [12,45,46]. Additionally, cardiac diseases are known to induce alterations in TH concentrations, which are correlated with increased in-hospital incidence and mortality rates of cardiac conditions [47].

Our study identified a significant correlation between the changes in LE8 and the levels of ln(TT3) and ln(Tg), with this relationship being notably pronounced within the normal range of TH concentrations. Using two domains and eight metrics that cover a variety of health behaviors and health factors such as blood pressure, blood glucose, blood lipids, and BMI, as well as health behaviors such as physical activity, diet, sleep hygiene, and nicotine exposure, LE8 provides a comprehensive tool for defining and quantifying cardiovascular health. Many studies have shown a strong correlation between TH levels and most health variables and behavioral indicators in the LE8 framework. A meta-analysis of 12 prospective observational studies demonstrated a significant association between abnormal TH levels and an increased risk of type 2 diabetes [48]. A comprehensive meta-analysis synthesized existing research on the relationship between subclinical hypothyroidism and sleep disorders and revealed a significant association between the two conditions [49]. In individuals with shorter sleep duration, an extension of sleep time correlated with a decrease in FT3 levels [50]. Moreover, dietary intake and weight-loss interventions are established factors that can significantly influence TH levels [51]. Our study established that LE8, which incorporates the aforementioned factors, is significantly correlated with TH. The study outcomes indicated that modifications in lifestyle and health factors associated with LE8 were associated with changes in TH levels in the general population, especially among those with normal TH levels.

TPOAb is the principal autoantibody against thyroid antigens, with increased levels typically observed in autoimmune thyroid diseases [52]. Our findings revealed a non-linear relationship between ln(TPOAb) and LE8 scores, with peak LE8 values observed within a moderate range of TPOAb levels. This suggests that favorable cardiovascular health profiles, as reflected by higher LE8 scores and encompassing healthy lifestyle factors and optimal metrics such as blood glucose, blood pressure, and lipid profiles, may be associated with physiologically balanced TPOAb levels (indicative of subclinical thyroid autoimmunity). Previous studies have explored the association between health factors and thyroid autoimmunity. For instance, smoking is correlated with the presence of TPOAb in the serum [53]. TPOAb positivity is more prevalent in patients with type 2 diabetes mellitus who exhibit visceral adiposity [54]. Our study revealed a potential nonlinear relationship between LE8 (a composite index encompassing these factors) and TPOAb levels, providing novel insights for future investigations into the interplay between thyroid autoimmunity and cardiovascular health.

Furthermore, our study underscores the invaluable role of LE8 score in fostering interdisciplinary collaboration [55], particularly between endocrinologists and cardiologists. By identifying lifestyle and health behavior factors associated with an increased risk of CVDs, the LE8 score serves not only as a tool for cardiovascular health assessment and management but also as a novel strategy for the improvement of thyroid health and potentially for the management of thyroid diseases. Adherence to LE8 recommendations, maintenance of a healthy lifestyle, and preservation of ideal health metrics may be useful for the prevention and management of thyroid diseases.

This study had several strengths. First, it is an inaugural exploration examining the correlation between TH and LE8 in the general U.S. adult population, offering a foundational reference for future investigations. Second, it leveraged extensive, high-quality data sourced from the NHANES, which is a comprehensive, ongoing cross-sectional study initiated in the 1980s that provides a representative sample of the civilian, non-institutionalized population in the U.S. Third, the study meticulously accounted for numerous potential covariates, including self-reported CVD, uric acid, creatinine, urine iodine, alcohol use, prescription drugs affecting thyroid function, and thyroid diseases, among others, thereby augmenting the robustness and reliability of the findings.

However, this epidemiological study has some limitations. First, owing to the cross-sectional design, definitive inferences regarding the direction or establishment of causality between TH and LE8 could not be ascertained. Although our findings demonstrate associations between FT3, FT4, TT3, TT4, Tg, TgAb, and TPOAb with LE8, our results underscore the necessity for longitudinal studies aimed at further investigating the impact of LE8 variations on TH levels. Additional investigations are required to establish causality. Second, despite the implementation of regression models and stratified analysis, the persistent influence of residual confounding factors due to unobserved or unrecognized variables could not be entirely mitigated. To bolster the validity of our results, we scrutinized the relationship between discrete TH groups and LE8, meticulously adjusting for a multitude of potential covariates in our analysis, thereby providing supplementary insights for future research. Third, given the limited sample size of the TH-abnormal group in the NHANES database, a comprehensive analysis of the association between abnormal TH levels and LE8 was not conducted. Instead, the association between TH and LE8 was examined in subgroups with adequate sample sizes, which revealed variations in the relationship between TH and LE8 across different levels of hormonal stratification. Finally, owing to the limitations in database completeness, the prescription medication records may potentially be incomplete. We exhaustively collected all the available prescription medication data. While data from 2007–2012 were included, we used the 2013–2018 NHANES Data Files containing ICD-10-CM diagnoses linked to prescription medications as the basis for extracting thyroid-related medications (including those for thyroid dysfunction, thyroid function interference, and thyroid diseases; see S1 Table). Additionally, thyroid-related prescription medications were systematically identified through a comprehensive review of published literature on the effects of pharmacological agents on thyroid function. Subsequently, the prescription medication records from 2007–2012 were extracted as covariates. Future research should include rigorously designed large-scale prospective cohort studies to reveal the causal relationship between TH and LE8. Additionally, an investigation into the underlying mechanisms of the association between TH and cardiovascular health is warranted.

## Conclusion

FT3, TT3, TT4, and Tg levels are associated with LE8. Nonlinear relationships were observed between Tg, TgAb, TPOAb, and LE8. Encouraging adherence to ideal lifestyle practices and maintaining health factors to improve LE8 scores may serve as a potential strategy for enhancing thyroid health. Furthermore, rigorously designed longitudinal studies are warranted to elucidate the causal relationship between cardiovascular health and TH levels.

## Supporting information

S1 Fig Visualization of Thyroid Hormones. (TIF)

S2 Fig Box Plots of Covariates in Post Hoc Analysis Stratified by life’s essential 8 Quartile Grouping. (TIF)

S3 Fig Box Plots of Covariates in Post Hoc Analysis Stratified by life’s essential 8 Quartile Grouping (Alt, Ast, and Alcohol User). (TIF)

S4 Fig Box Plots of TT4 and Tg in Post Hoc Analysis Stratified by life’s essential 8 Quartile Grouping. (TIF)

S5 Fig Box Plots of Thyroid Hormones in Post Hoc Analysis Stratified by life’s essential 8 Quartile Grouping. (TIF)

S6 Fig Box Plots of ln(TT4) and ln(Tg) in Post Hoc Analysis Stratified by life’s essential 8 Quartile Grouping. (TIF)

S7 Fig Box Plots of ln(TSH), ln(FT3), ln(FT4),ln(TT3), ln(TgAb),ln(TPOAb) in Post Hoc Analysis Stratified by life’s essential 8 Quartile Grouping. (TIF)

S8 Fig Graphical summary of fully adjusted linear regression model for ln(TSH) and life’s essential 8. (TIF)

S9 Fig Graphical summary of fully adjusted linear regression model for ln(FT3) and life’s essential 8. (TIF)

S10 Fig Graphical summary of fully adjusted linear regression model for ln(FT4) and life’s essential 8. (TIF)

S11 Fig Graphical summary of fully adjusted linear regression model for ln(TT3) and life’s essential 8. (TIF)

S12 Fig Graphical summary of fully adjusted linear regression model for ln(TT4) and life’s essential 8. (TIF)

S13 Fig Graphical summary of fully adjusted linear regression model for ln(Tg) and life’s essential 8. (TIF)

S14 Fig Graphical summary of fully adjusted linear regression model for ln(TgAb) and life’s essential 8. (TIF)

S15 Fig Graphical summary of fully adjusted linear regression model for ln(TPOAb) and life’s essential 8. (TIF)

S16 Fig Restricted cubic spline plots between ln(Tg), ln(TgAb), ln(TPOAb), and life’s essential 8 in cardiovascular disease-free cohort. (TIF)

S17 Fig Restricted cubic spline plots between ln(Tg), ln(TgAb), ln(TPOAb), and life’s essential 8 in thyroid disease-free cohort. (TIF)

S18 Fig Restricted cubic spline plots between ln(Tg), ln(TgAb), ln(TPOAb), and life’s essential 8 under SAF weight and missing data deletion. (TIF)

S19 Fig Restricted cubic spline plots between ln(Tg), ln(TgAb), ln(TPOAb), and life’s essential 8 under MEC weight and single imputation for missing data. (TIF)

S20 Fig Restricted cubic spline plots between ln(Tg), ln(TgAb), ln(TPOAb), and life’s essential 8 under MEC weight and missing data deletion. (TIF)

S1 Table Corresponding Prescription Medications Names and code in NHANES. (DOCX)

S2 Table Selection of covariates and analysis of collinearity in overall patients. (DOCX)

S3 Table Association of covariates and life’s essential 8. (DOCX)

S4 Table Association between thyroid hormone and life’s essential 8 after excluding specific populations. (DOCX)

S5 Table Association between thyroid hormone and life’s essential 8 under different missing data handling and weighting methods. (DOCX)

S1 Data Database access link. (DOCX)

## Abbreviations

LE8: Life’s essential 8
TSH: Thyroid stimulating hormone
T3: Triiodothyronine
T4: Thyroxine
FT3: free triiodothyronine
FT4: free thyroxine
TT3: total triiodothyronine
TT4: total thyroxine
Tg: Thyroglobulin
TgAb: Thyroglobulin antibodies
TPOAb: Thyroid peroxidase antibody
TH: Thyroid Hormone
CVD: Cardiovascular disease
AHA: American Heart Association
LS7: Life’s Simple 7
U.S.: United States
NHANES: National Health and Nutrition Examination Survey
NCHS: National Center for Health Statistics
ICD-10-CM: International Classification of Diseases-Tenth Revision-Clinical Modification
RCS: Restricted cubic spline
BMI: Body mass index
DASH: Dietary Approaches to Stop Hypertension
Alt: Alanine aminotransferase
Ast: Aspartate aminotransferase
PIR: Poverty income ratio
MEC: Mobile Examination Center
WTSAF2YR: Fasting subsample 2 year Mec weight
