# Supplementary figures and images for: Association between thyroid hormone and cardiovascular health: A cross-sectional study
Source: PLoS One. 2025 Oct 24;20(10):e0329194. doi: 10.1371/journal.pone.0329194 (PMC12551862; doi:10.1371/journal.pone.0329194)

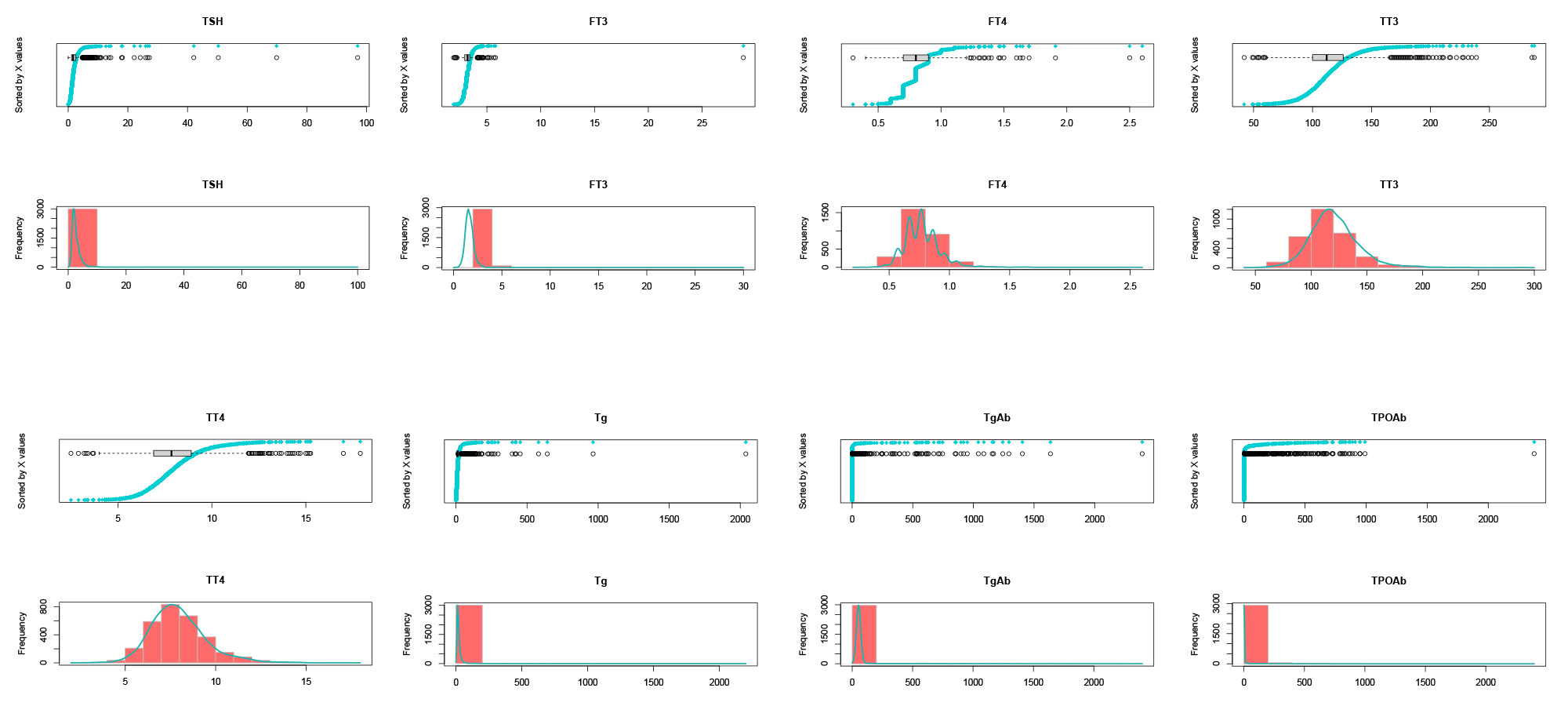

Supplement: S1 Fig — (TIF) [file pone.0329194.s001.tif]

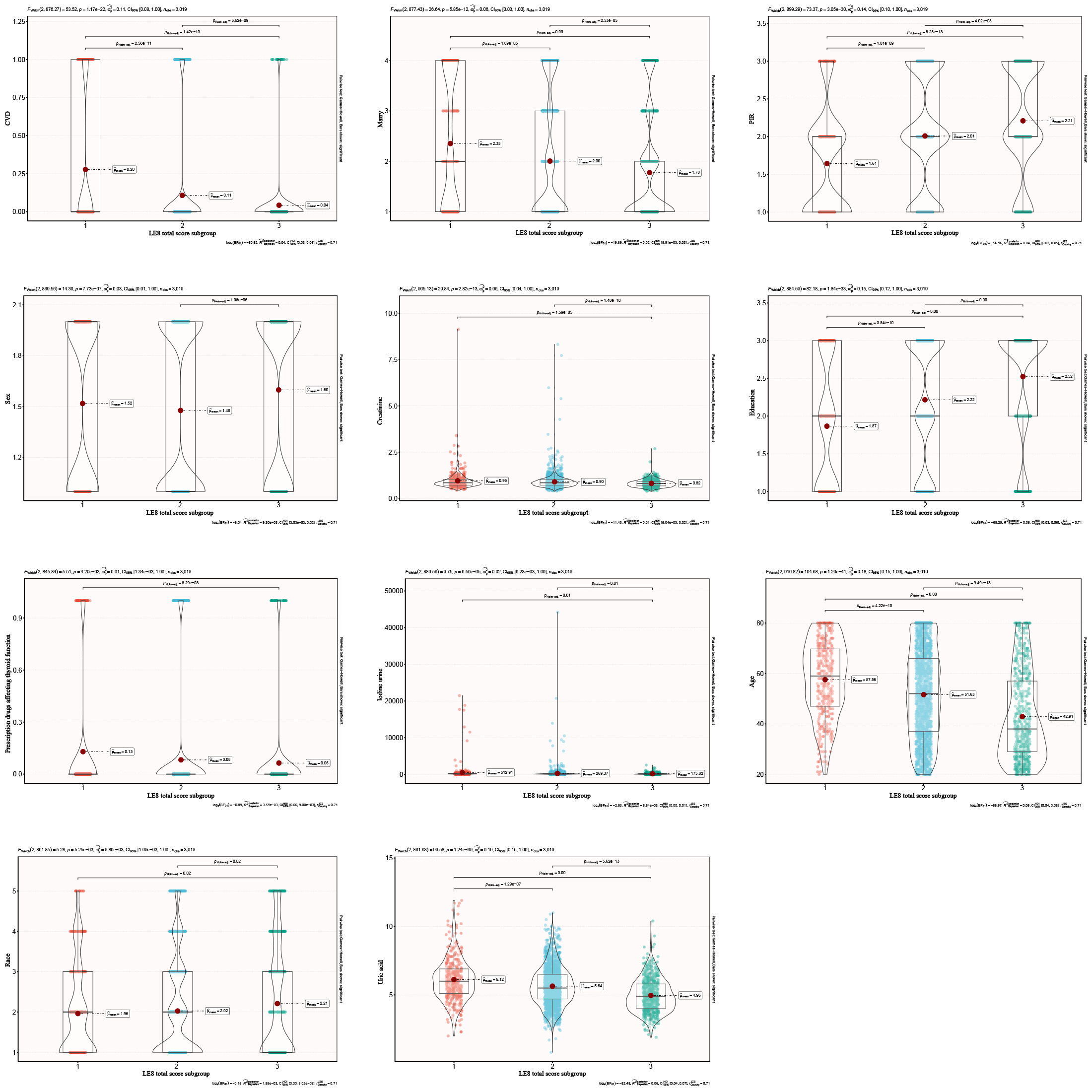

Supplement: S2 Fig — (TIF) [file pone.0329194.s002.tif]

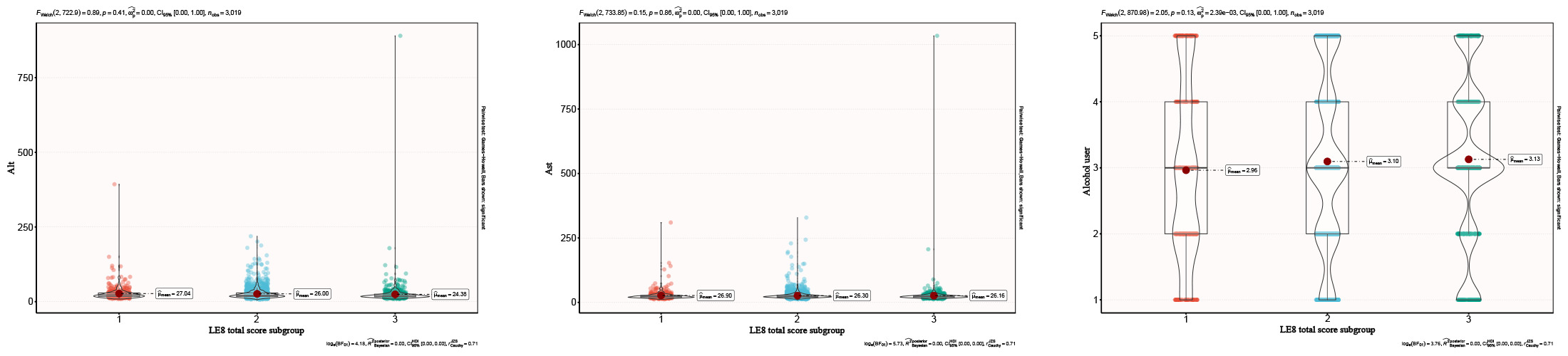

Supplement: S3 Fig — (TIF) [file pone.0329194.s003.tif]

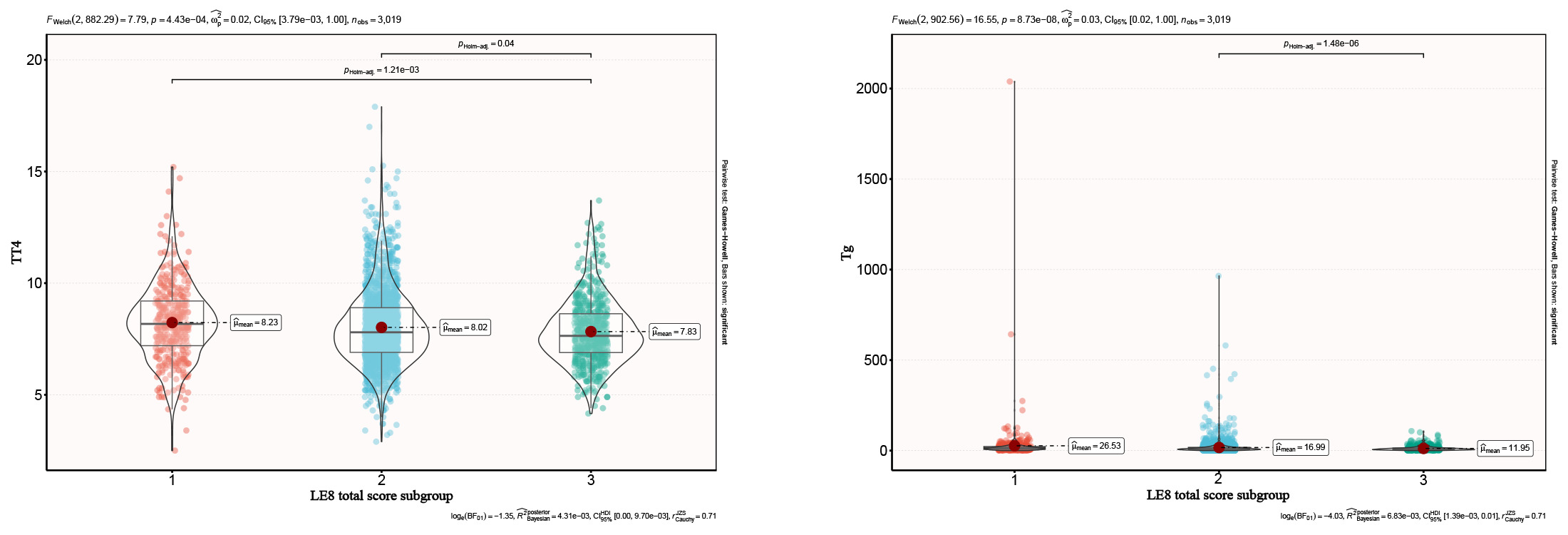

Supplement: S4 Fig — (TIF) [file pone.0329194.s004.tif]

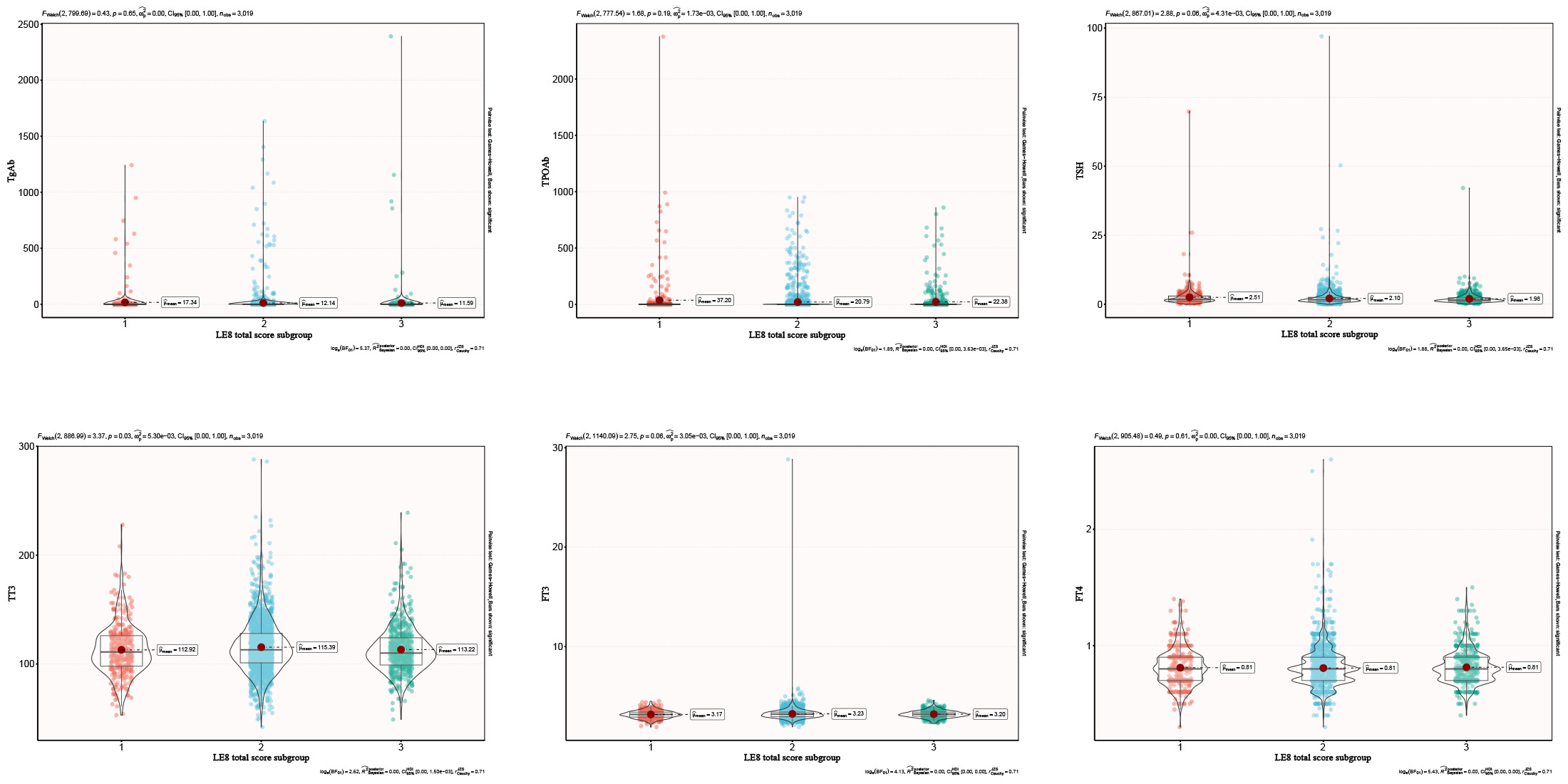

Supplement: S5 Fig — (TIF) [file pone.0329194.s005.tif]

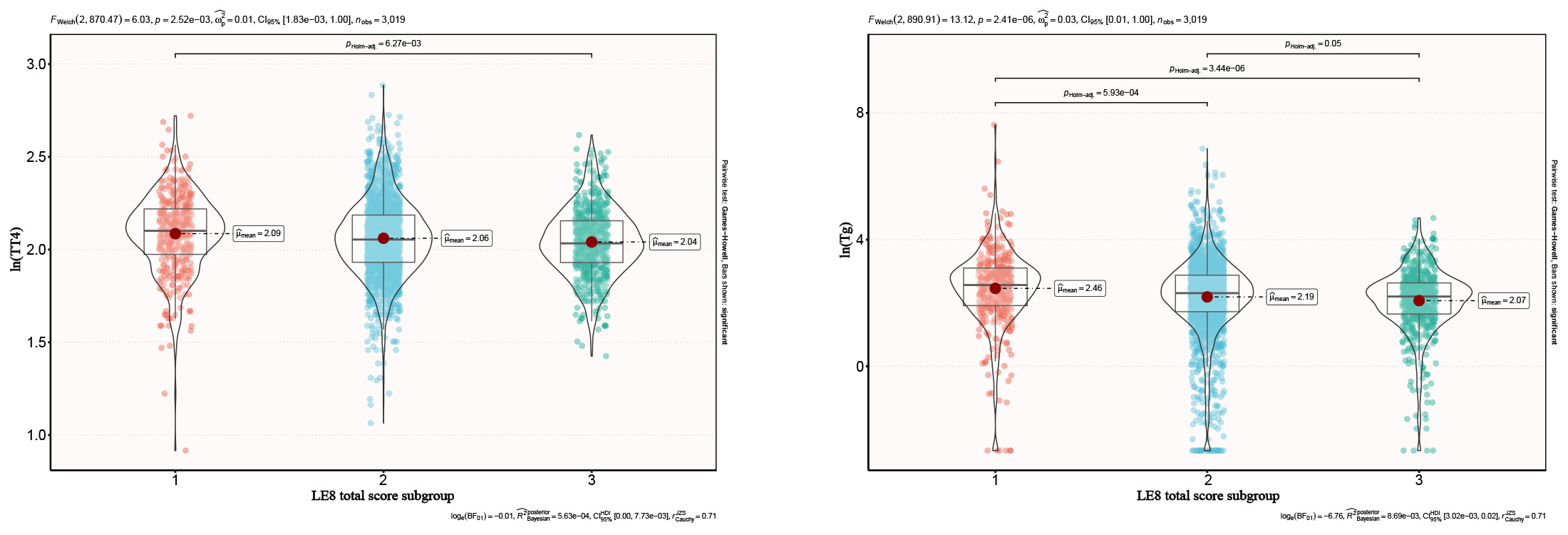

Supplement: S6 Fig — (TIF) [file pone.0329194.s006.tif]

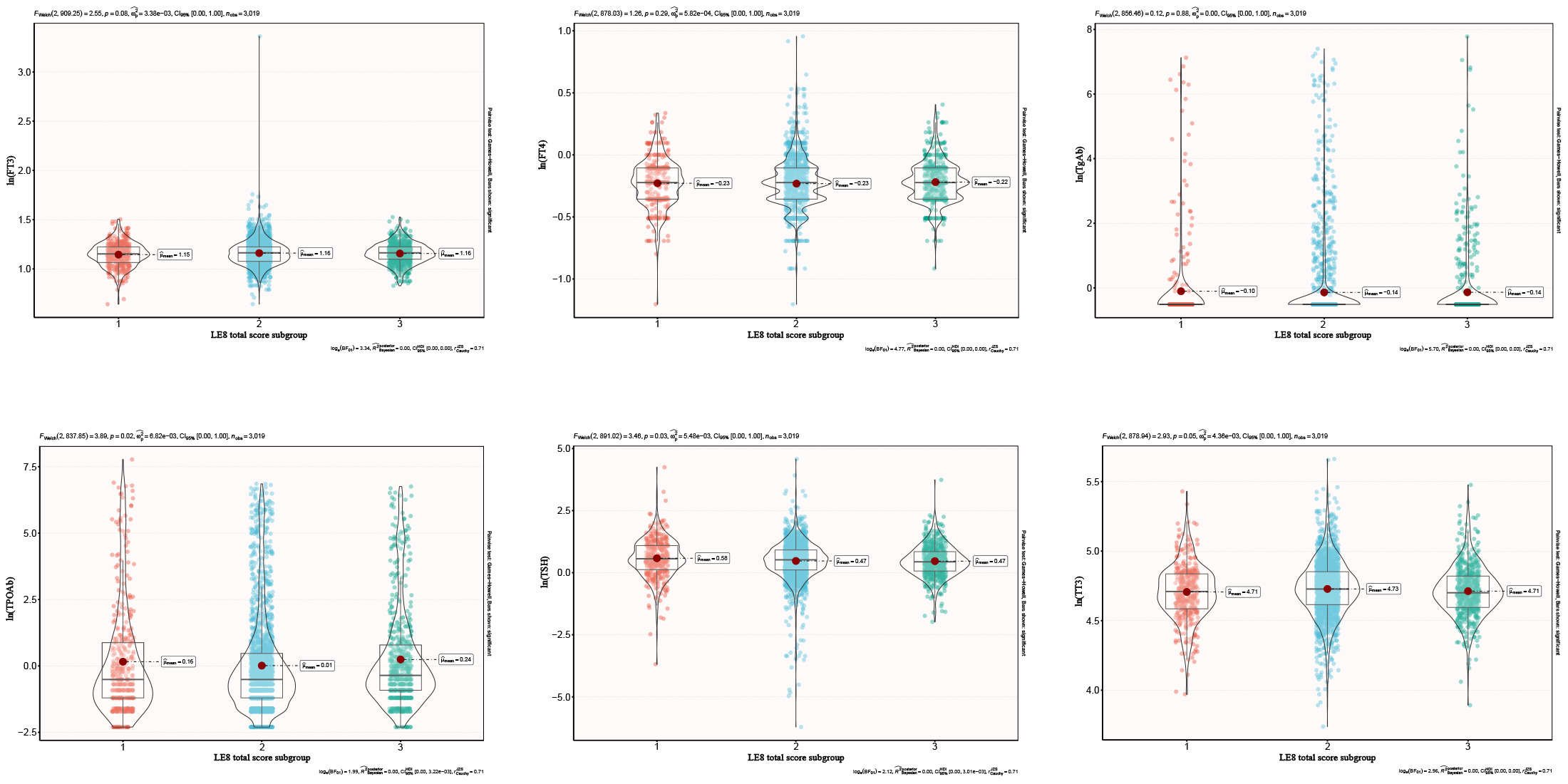

Supplement: S7 Fig — (TIF) [file pone.0329194.s007.tif]

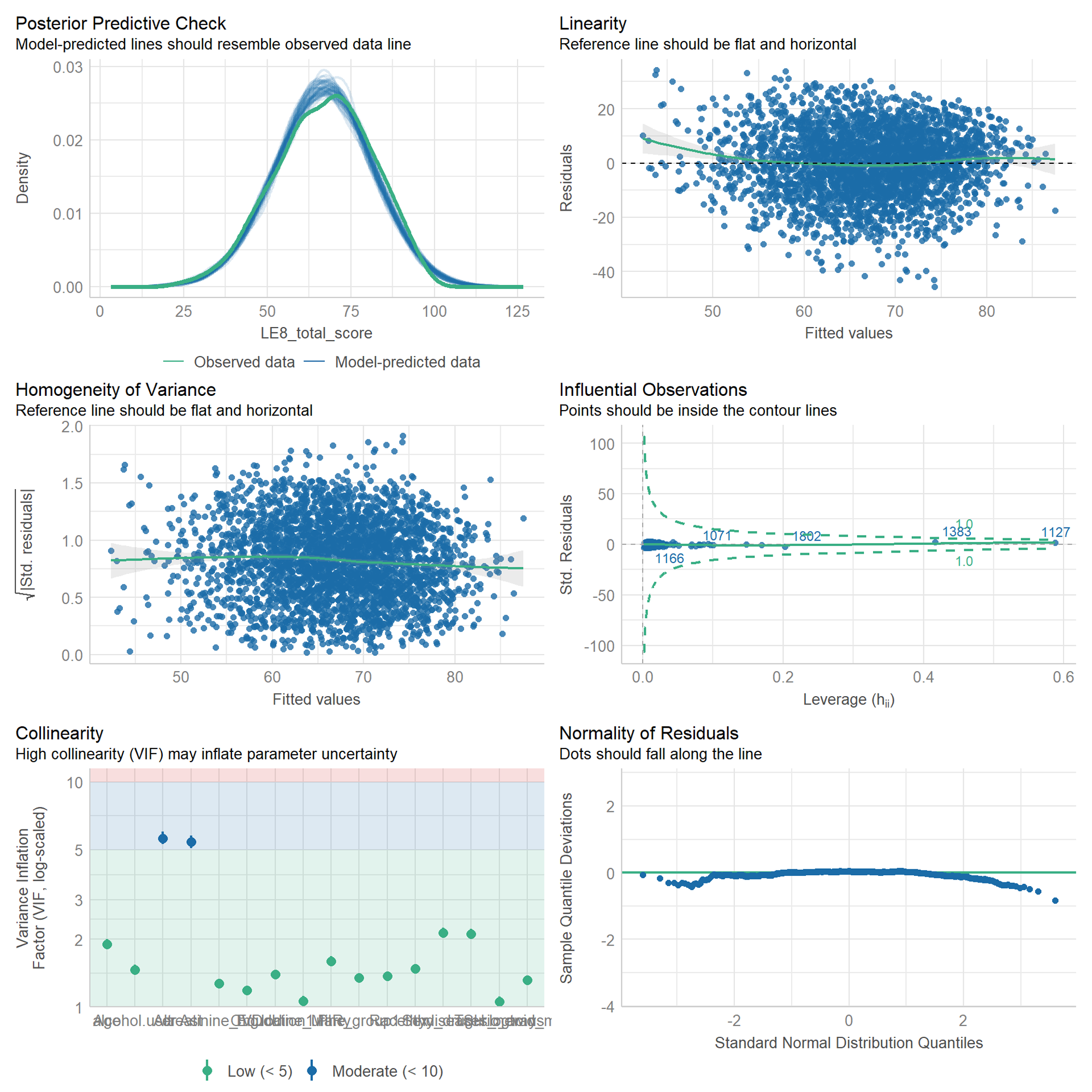

Supplement: S8 Fig — (TIF) [file pone.0329194.s008.tif]

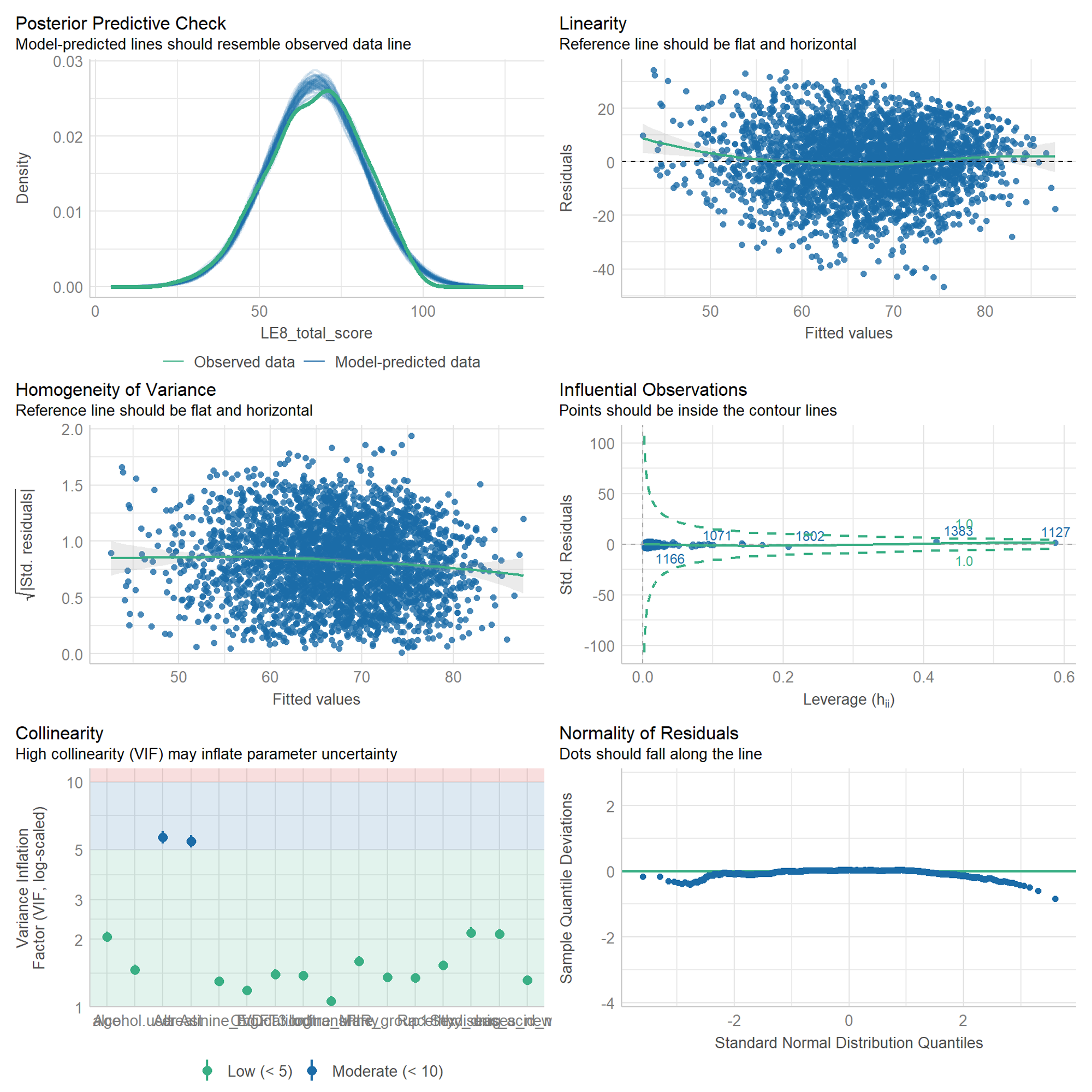

Supplement: S9 Fig — (TIF) [file pone.0329194.s009.tif]

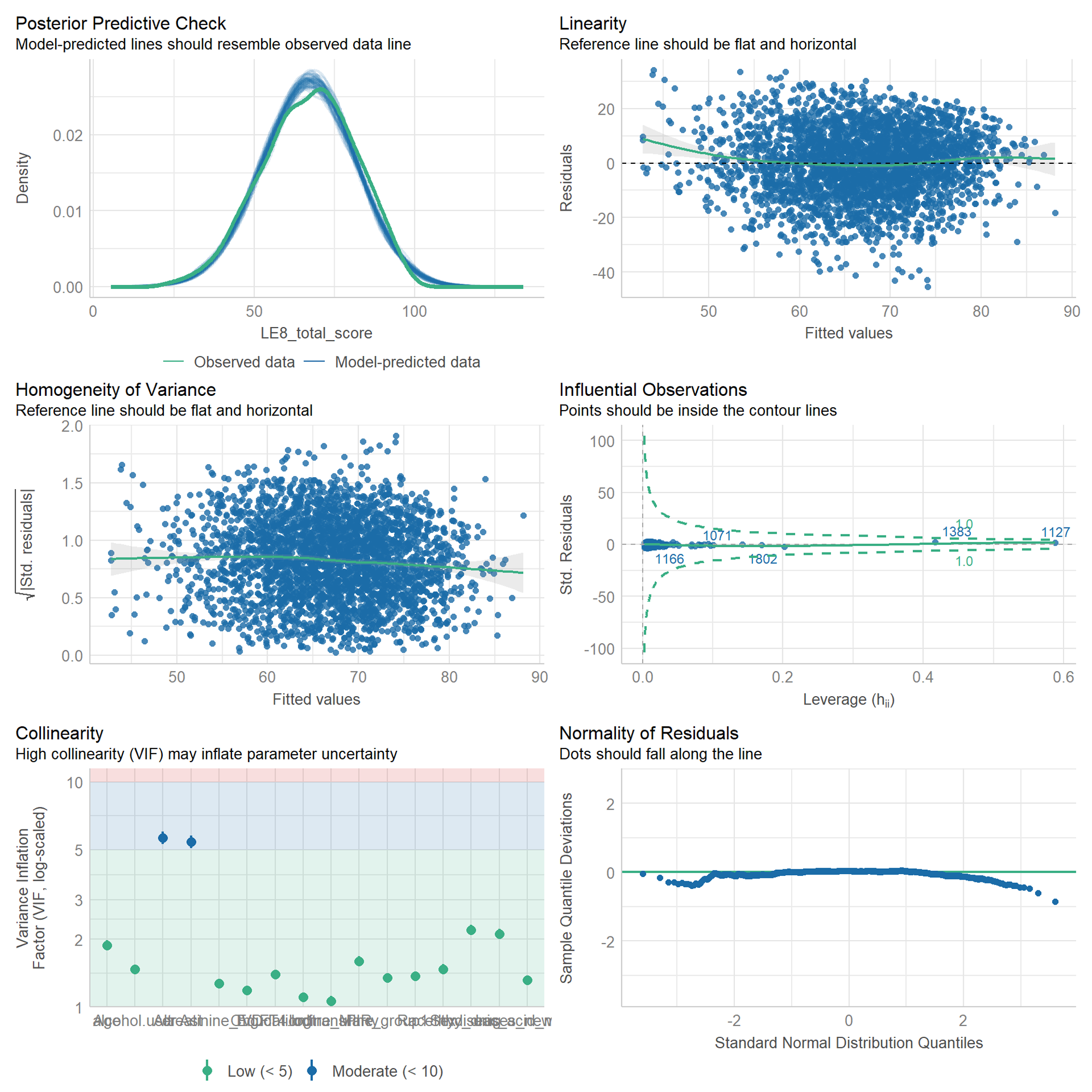

Supplement: S10 Fig — (TIF) [file pone.0329194.s010.tif]

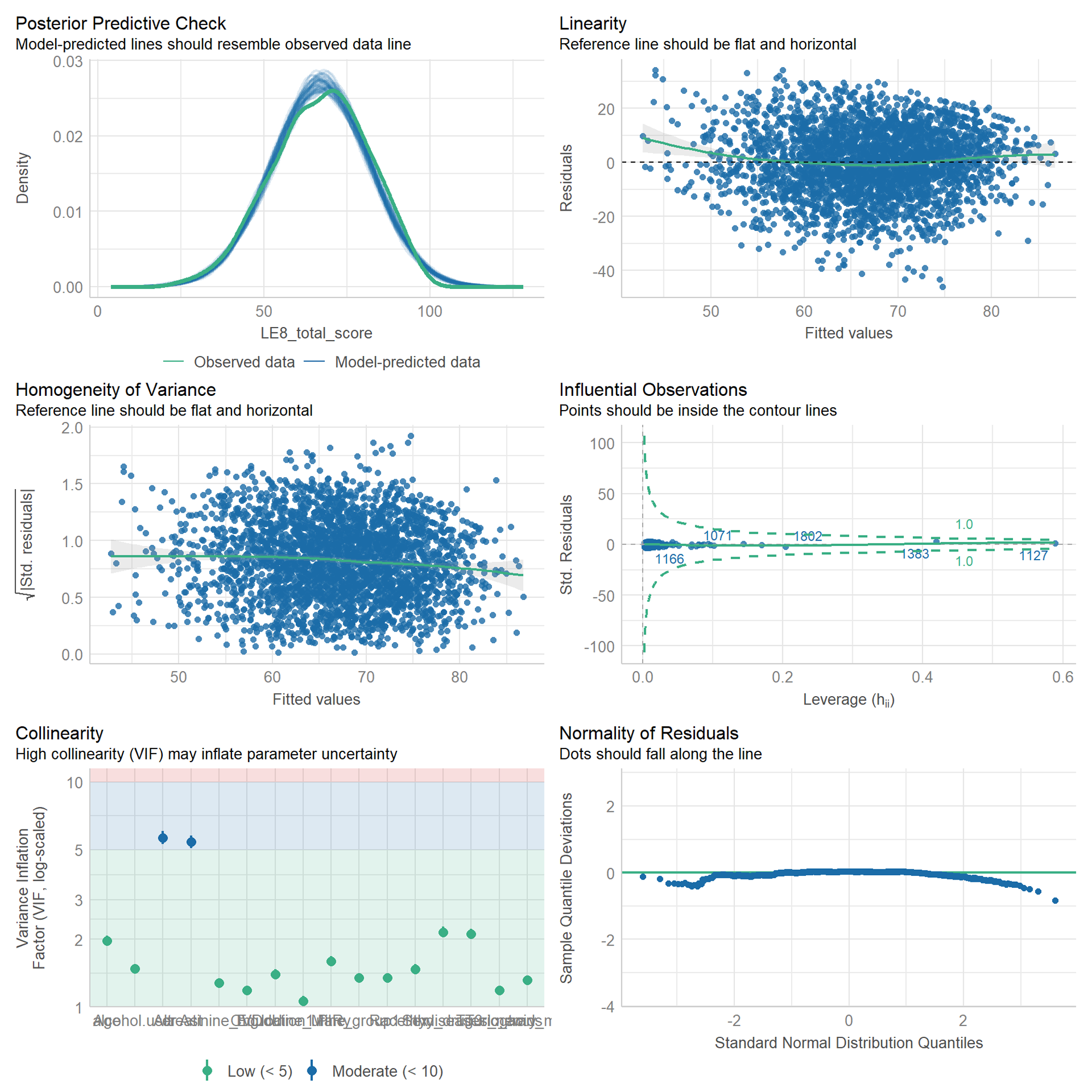

Supplement: S11 Fig — (TIF) [file pone.0329194.s011.tif]

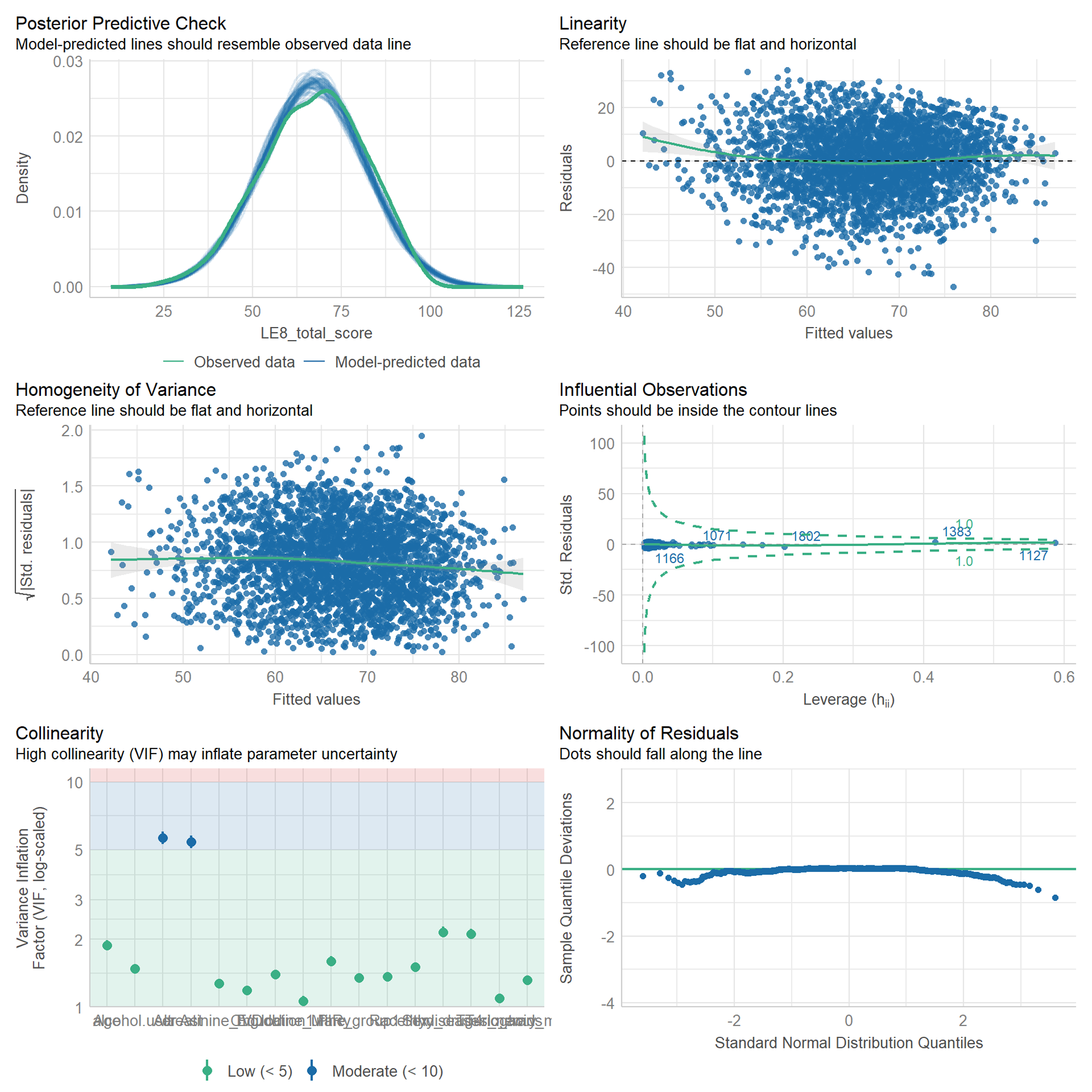

Supplement: S12 Fig — (TIF) [file pone.0329194.s012.tif]

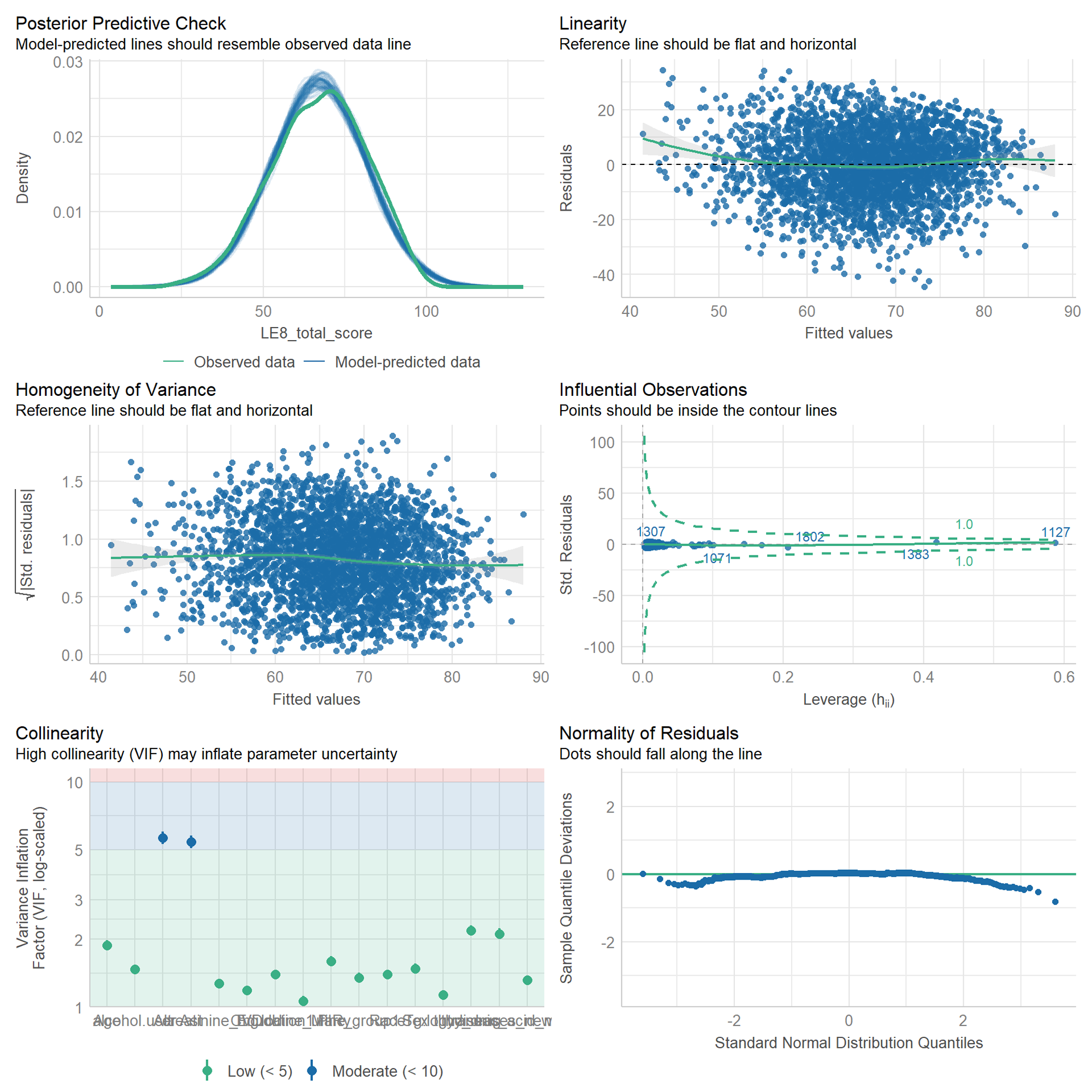

Supplement: S13 Fig — (TIF) [file pone.0329194.s013.tif]

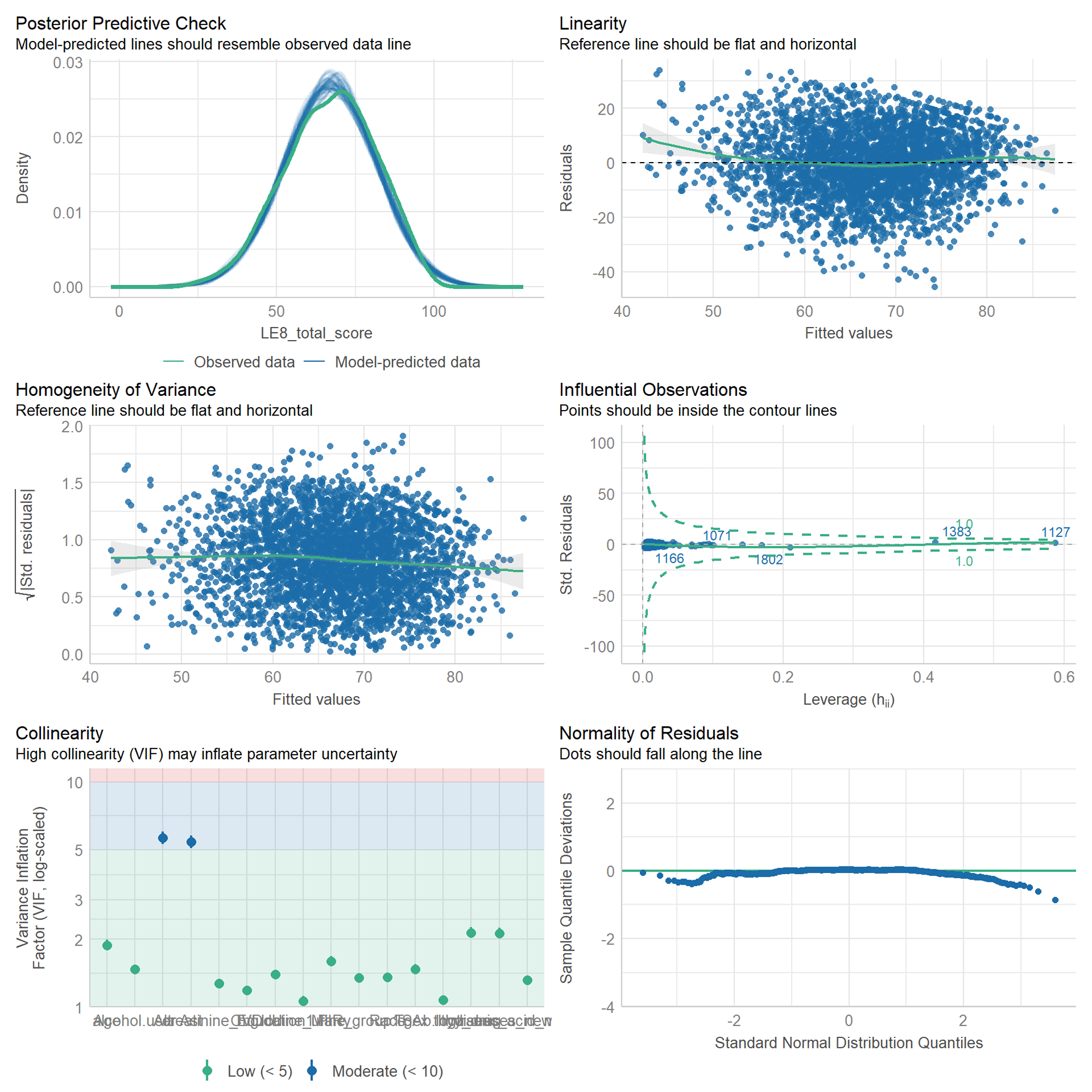

Supplement: S14 Fig — (TIF) [file pone.0329194.s014.tif]

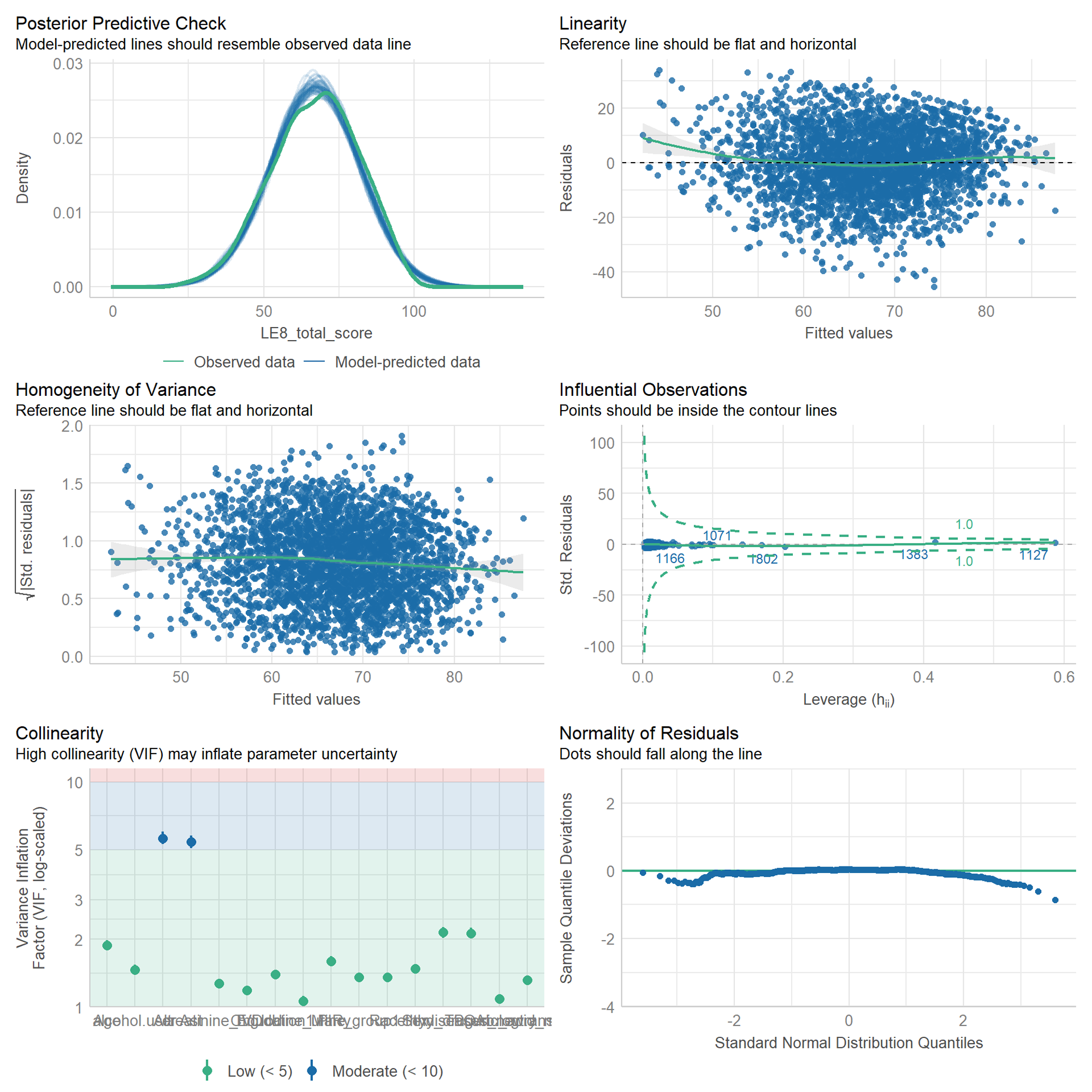

Supplement: S15 Fig — (TIF) [file pone.0329194.s015.tif]

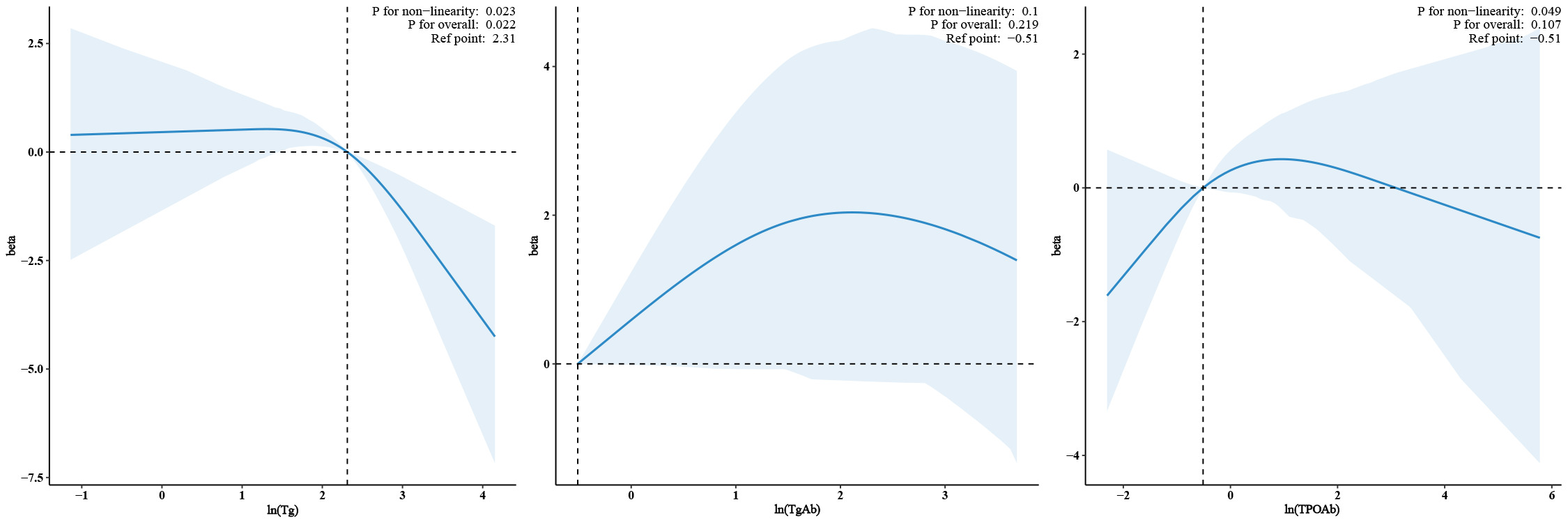

Supplement: S16 Fig — (TIF) [file pone.0329194.s016.tif]

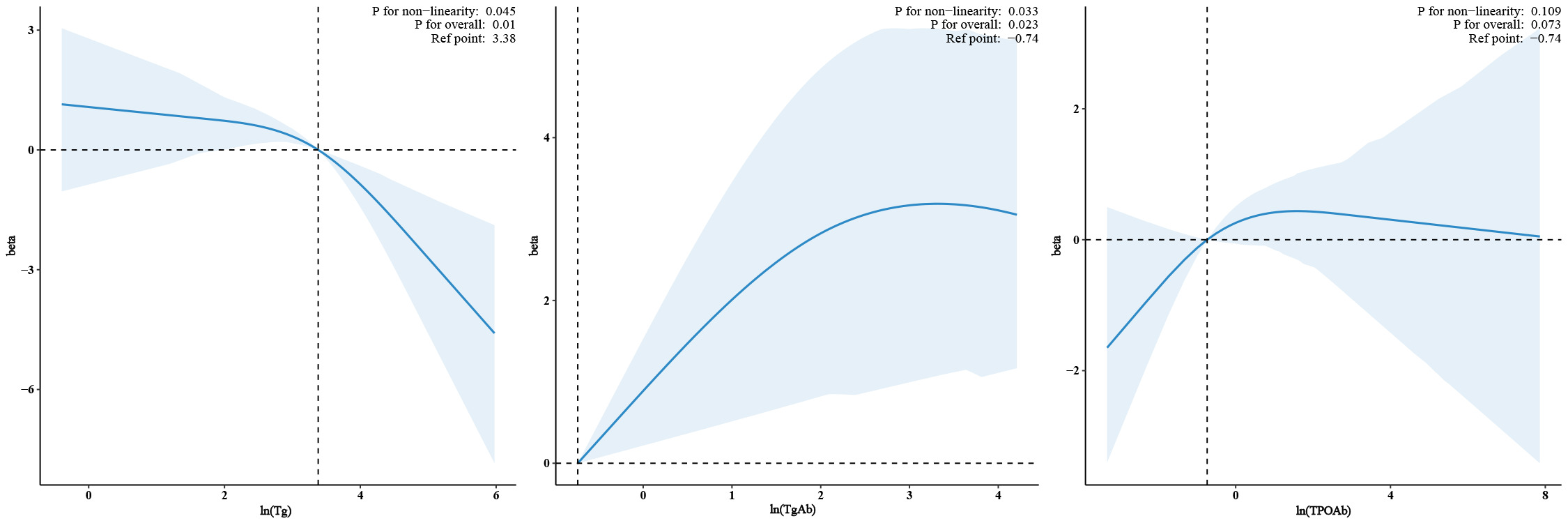

Supplement: S17 Fig — (TIF) [file pone.0329194.s017.tif]

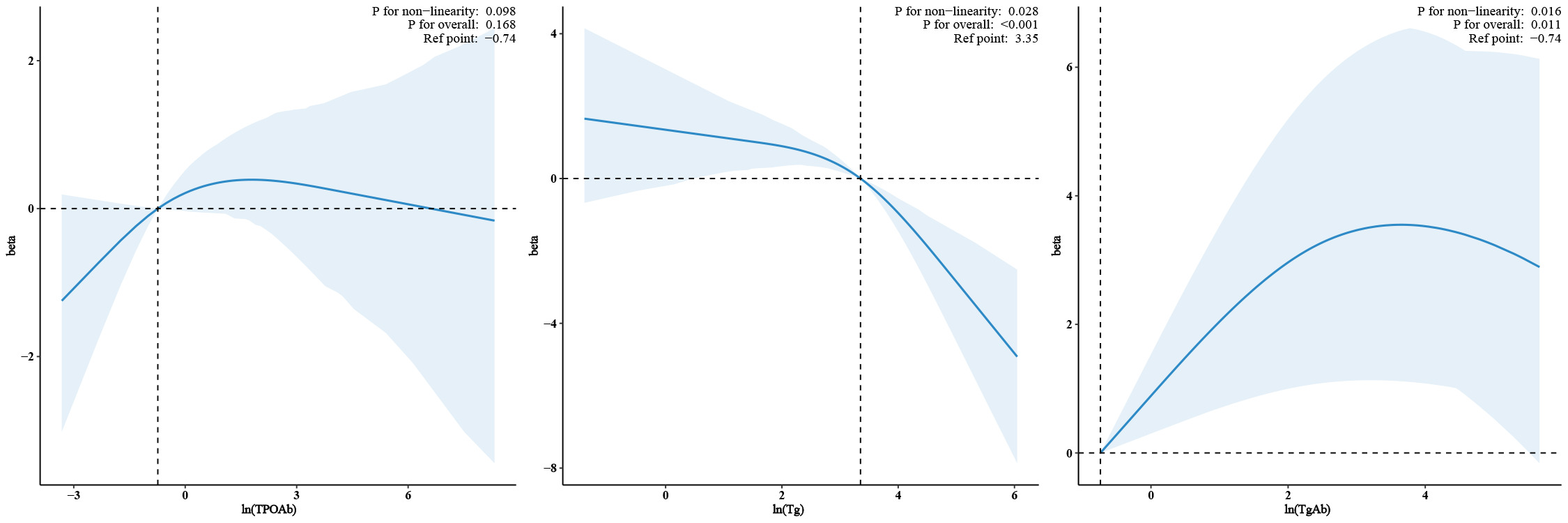

Supplement: S18 Fig — (TIF) [file pone.0329194.s018.tif]

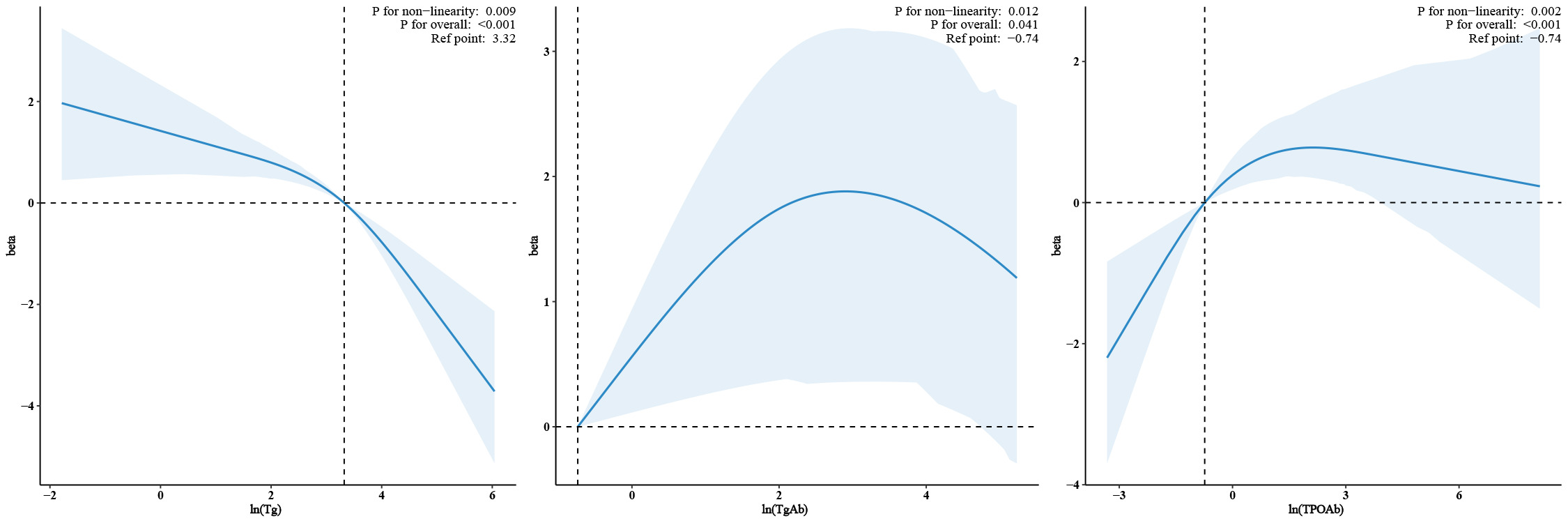

Supplement: S19 Fig — (TIF) [file pone.0329194.s019.tif]

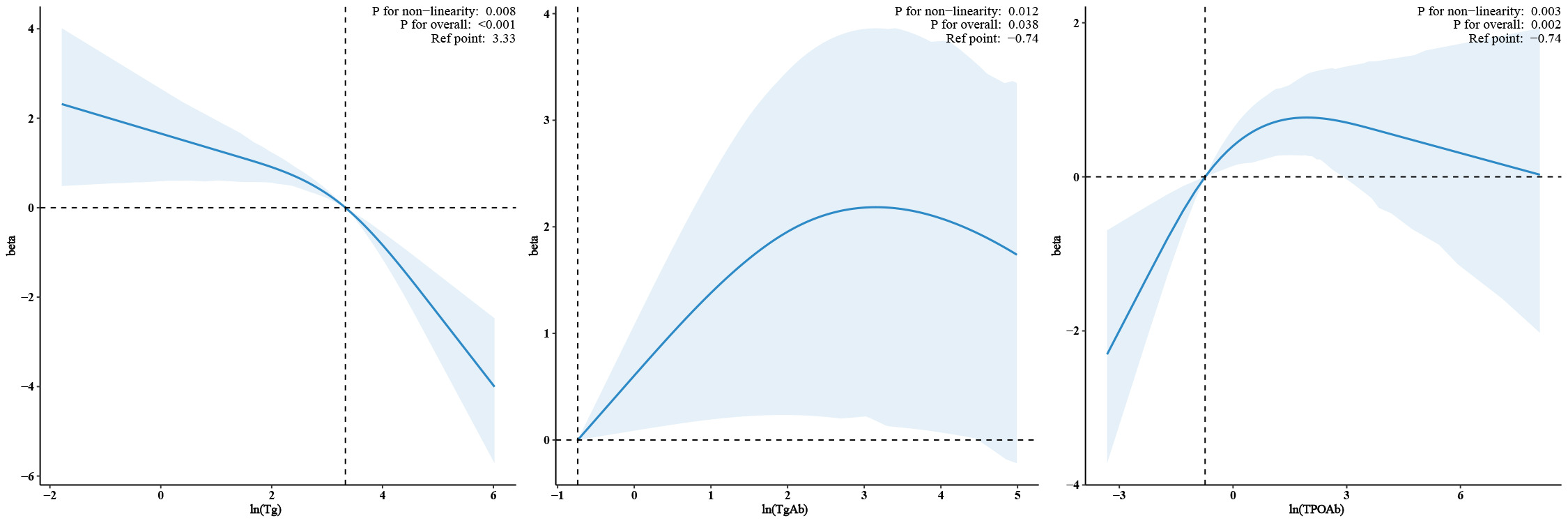

Supplement: S20 Fig — (TIF) [file pone.0329194.s020.tif]
