# Supplementary material for: Association between thyroid hormone and cardiovascular health: A cross-sectional study
Source: PLoS One. 2025 Oct 24;20(10):e0329194. doi: 10.1371/journal.pone.0329194 (PMC12551862; doi:10.1371/journal.pone.0329194)
Supplement: S1 Table — (DOCX) [file pone.0329194.s021.docx]

**S1 Table. Corresponding Standardized Generic Prescription Medication Names in NHANES.**

| **Information** | **Drug code** | **Drug name** |
| --- | --- | --- |
| Prescription medications associated with thyroid dysfunction, interference with thyroid function, and thyroid disease | d00278 | LEVOTHYROXINE |
|  | h00019 | LEVOTHYROXINE; LIOTHYRONINE |
|  | d00658 | LIOTHYRONINE |
|  | d00290 | METHIMAZOLE |
|  | d00655 | THYROID DESICCATED |
|  | a55820 | THYROGLOBULIN |
|  | c00103 | THYROID HORMONES - UNSPECIFIED |
|  | d00361 | PROPYLTHIOURACIL |
|  | d04112 | CABERGOLINE |
|  | d00254 | HYDROCORTISONE |
|  | d00350 | PREDNISONE |
|  | d04205 | POVIDONE IODINE OPHTHALMIC |
|  | d00002 | AMIODARONE |
|  | d00178 | BROMOCRIPTINE |
|  | d00061 | LITHIUM |
