# Supplementary material for: Association between thyroid hormone and cardiovascular health: A cross-sectional study
Source: PLoS One. 2025 Oct 24;20(10):e0329194. doi: 10.1371/journal.pone.0329194 (PMC12551862; doi:10.1371/journal.pone.0329194)
Supplement: S2 Table — (DOCX) [file pone.0329194.s022.docx]

**S2 Table. Selection of covariates and analysis of collinearity in overall patients.**

| **Variable** | **Term** | **Forward selection** | | **Backward elimination** | | **GVIF** | **DF** | **GVIF^(1/(2*Df))** | **Colinearity** |
| --- | --- | --- | --- | --- | --- | --- | --- | --- | --- |
|  |  | **Coefficient** | **Change percentage** | **Coefficient** | **Change percentage** |  |  |  |  |
| ln(TSH) | Crude/Full | -0.95 | Ref. | -0.47 | Ref. | 1.053 | 1 | 1.026 | 0 |
|  | Age | -0.26 | -73.1 | -0.81 | 71.8 | 1.901 | 1 | 1.379 | 0 |
|  | Sex | -0.97 | 2.1 | -0.51 | 8.0 | 1.476 | 1 | 1.215 | 0 |
|  | Race | -1.21 | 27.6 | -0.17 | -63.9 | 1.37 | 4 | 1.040 | 0 |
|  | Marry | -0.87 | -8.0 | -0.47 | -0.2 | 1.598 | 3 | 1.081 | 0 |
|  | PIR | -1.00 | 5.5 | -0.44 | -7.7 | 1.348 | 2 | 1.078 | 0 |
|  | Education | -0.83 | -12.7 | -0.54 | 13.7 | 1.395 | 2 | 1.087 | 0 |
|  | Alcohol user | -0.88 | -7.8 | -0.45 | -4.5 | 1.466 | 4 | 1.049 | 0 |
|  | CVD | -0.91 | -4.7 | -0.40 | -16.5 | 1.187 | 1 | 1.089 | 0 |
|  | Alt | -0.97 | 1.7 | -0.46 | -3.2 | 5.632 | 1 | 2.373 | 1 |
|  | Ast | -0.95 | 0.3 | -0.47 | -0.9 | 5.422 | 1 | 2.328 | 1 |
|  | Creatinine | -0.89 | -6.6 | -0.47 | -0.9 | 1.269 | 1 | 1.126 | 0 |
|  | Uric acid | -0.72 | -24.3 | -0.67 | 41 | 1.317 | 1 | 1.148 | 0 |
|  | Iodine urine | -0.89 | -6.3 | -0.5 | 5.4 | 1.064 | 1 | 1.032 | 0 |
|  | Prescription drugs affecting thyroid function | -0.99 | 3.8 | -0.47 | -1.3 | 2.134 | 1 | 1.461 | 0 |
|  | Thyroid diseases | -0.97 | 1.8 | -0.47 | -0.7 | 2.11 | 1 | 1.453 | 0 |
| ln(FT3) | Crude/Full | 3.55 | Ref. | -6.88 | Ref. | 1.379 | 1 | 1.174 | 0 |
|  | Age | -12.11 | -441.4 | -0.29 | -95.8 | 2.048 | 1 | 1.431 | 0 |
|  | Sex | 4.92 | 38.8 | -5.61 | -18.4 | 1.531 | 1 | 1.237 | 0 |
|  | Race | 2.27 | -35.9 | -6.03 | -12.4 | 1.347 | 4 | 1.038 | 0 |
|  | Marry | 0.65 | -81.8 | -7.15 | 3.9 | 1.598 | 3 | 1.081 | 0 |
|  | PIR | 6.25 | 76.4 | -8.14 | 18.3 | 1.355 | 2 | 1.079 | 0 |
|  | Education | 4.82 | 36.0 | -7.71 | 12.1 | 1.400 | 2 | 1.088 | 0 |
|  | Alcohol user | 3.17 | -10.6 | -6.85 | -0.5 | 1.466 | 4 | 1.049 | 0 |
|  | CVD | -0.69 | -119.6 | -6.51 | -5.3 | 1.186 | 1 | 1.089 | 0 |
|  | Alt | 4.09 | 15.2 | -7.96 | 15.6 | 5.668 | 1 | 2.381 | 1 |
|  | Ast | 3.58 | 0.9 | -7.75 | 12.6 | 5.448 | 1 | 2.334 | 1 |
|  | Creatinine | 1.18 | -66.6 | -7.08 | 2.9 | 1.300 | 1 | 1.140 | 0 |
|  | Uric acid | 4.60 | 29.8 | -8.14 | 18.3 | 1.317 | 1 | 1.147 | 0 |
|  | Iodine urine | 3.02 | -14.8 | -6.76 | -1.8 | 1.061 | 1 | 1.030 | 0 |
|  | Prescription drugs affecting thyroid function | 1.88 | -47.1 | -6.80 | -1.2 | 2.139 | 1 | 1.462 | 0 |
|  | Thyroid diseases | 2.70 | -23.7 | -6.90 | 0.2 | 2.110 | 1 | 1.453 | 0 |
| ln(FT4) | Crude/Full | 0.34 | Ref. | 1.35 | Ref. | 1.108 | 1 | 1.053 | 0 |
|  | Age | 2.22 | 552.6 | 0.94 | -30.5 | 1.881 | 1 | 1.371 | 0 |
|  | Sex | 0.28 | -16.6 | 1.40 | 3.7 | 1.473 | 1 | 1.214 | 0 |
|  | Race | -1.26 | -469.6 | 2.48 | 83.1 | 1.372 | 4 | 1.040 | 0 |
|  | Marry | 0.20 | -42.6 | 1.40 | 3.3 | 1.599 | 3 | 1.081 | 0 |
|  | PIR | 0.54 | 59.4 | 1.12 | -17.2 | 1.348 | 2 | 1.078 | 0 |
|  | Education | 0.15 | -55.6 | 1.52 | 12.7 | 1.394 | 2 | 1.087 | 0 |
|  | Alcohol user | 0.64 | 88.4 | 1.41 | 4.5 | 1.467 | 4 | 1.049 | 0 |
|  | CVD | 1.46 | 328.8 | 1.19 | -12.4 | 1.185 | 1 | 1.089 | 0 |
|  | Alt | 0.21 | -39.6 | 1.71 | 26.8 | 5.641 | 1 | 2.375 | 1 |
|  | Ast | 0.32 | -4.9 | 1.6 | 18.2 | 5.427 | 1 | 2.33 | 1 |
|  | Creatinine | 0.72 | 111.4 | 1.39 | 2.4 | 1.269 | 1 | 1.127 | 0 |
|  | Uric acid | 0.59 | 72.5 | 1.26 | -7.0 | 1.313 | 1 | 1.146 | 0 |
|  | Iodine urine | 0.58 | 69.4 | 1.34 | -0.8 | 1.06 | 1 | 1.029 | 0 |
|  | Prescription drugs affecting thyroid function | 1.98 | 481.4 | 1.23 | -8.8 | 2.195 | 1 | 1.481 | 0 |
|  | Thyroid diseases | 1.07 | 215.4 | 1.35 | -0.5 | 2.11 | 1 | 1.452 | 0 |
| ln(TT3) | Crude/Full | 1.01 | Ref. | -3.88 | Ref. | 1.187 | 1 | 1.089 | 0 |
|  | Age | -5.65 | -659.2 | -1.03 | -73.3 | 1.967 | 1 | 1.402 | 0 |
|  | Sex | 1.22 | 20.4 | -3.78 | -2.4 | 1.474 | 1 | 1.214 | 0 |
|  | Race | 0.44 | -56.7 | -3.51 | -9.4 | 1.348 | 4 | 1.038 | 0 |
|  | Marry | -0.37 | -136.9 | -3.83 | -1.2 | 1.595 | 3 | 1.081 | 0 |
|  | PIR | 2.31 | 128.5 | -4.3 | 10.9 | 1.35 | 2 | 1.078 | 0 |
|  | Education | 2.10 | 107.9 | -4.52 | 16.6 | 1.399 | 2 | 1.088 | 0 |
|  | Alcohol user | 1.13 | 12.0 | -3.7 | -4.5 | 1.478 | 4 | 1.050 | 0 |
|  | CVD | -1.26 | -224.9 | -3.68 | -5.1 | 1.185 | 1 | 1.089 | 0 |
|  | Alt | 1.34 | 32.8 | -4.46 | 14.9 | 5.661 | 1 | 2.379 | 1 |
|  | Ast | 1.05 | 4.3 | -4.20 | 8.2 | 5.432 | 1 | 2.331 | 1 |
|  | Creatinine | -0.40 | -139.6 | -3.98 | 2.7 | 1.283 | 1 | 1.132 | 0 |
|  | Uric acid | 0.86 | -14.6 | -4.55 | 17.3 | 1.316 | 1 | 1.147 | 0 |
|  | Iodine urine | 0.63 | -37.9 | -3.79 | -2.4 | 1.062 | 1 | 1.031 | 0 |
|  | Prescription drugs affecting thyroid function | -0.11 | -111.3 | -3.80 | -1.9 | 2.148 | 1 | 1.466 | 0 |
|  | Thyroid diseases | 0.48 | -52.3 | -3.88 | 0 | 2.110 | 1 | 1.452 | 0 |
| ln(TT4) | Crude/Full | -5.97 | Ref. | -3.50 | Ref. | 1.093 | 1 | 1.046 | 0 |
|  | Age | -4.52 | -24.3 | -3.58 | 2.2 | 1.879 | 1 | 1.371 | 0 |
|  | Sex | -6.68 | 12.0 | -3.84 | 9.7 | 1.503 | 1 | 1.226 | 0 |
|  | Race | -6.83 | 14.5 | -2.69 | -23.2 | 1.36 | 4 | 1.039 | 0 |
|  | Marry | -5.51 | -7.6 | -3.45 | -1.5 | 1.596 | 3 | 1.081 | 0 |
|  | PIR | -5.10 | -14.5 | -3.73 | 6.5 | 1.348 | 2 | 1.078 | 0 |
|  | Education | -4.96 | -16.9 | -3.70 | 5.8 | 1.394 | 2 | 1.087 | 0 |
|  | Alcohol user | -5.20 | -12.9 | -3.50 | -0.2 | 1.477 | 4 | 1.050 | 0 |
|  | CVD | -5.34 | -10.5 | -3.57 | 2 | 1.185 | 1 | 1.088 | 0 |
|  | Alt | -5.94 | -0.4 | -3.64 | 4 | 5.633 | 1 | 2.373 | 1 |
|  | Ast | -5.95 | -0.2 | -3.53 | 0.9 | 5.422 | 1 | 2.328 | 1 |
|  | Creatinine | -5.87 | -1.7 | -3.45 | -1.6 | 1.271 | 1 | 1.127 | 0 |
|  | Uric acid | -5.77 | -3.2 | -4.41 | 25.9 | 1.319 | 1 | 1.149 | 0 |
|  | Iodine urine | -5.84 | -2.1 | -3.51 | 0.1 | 1.06 | 1 | 1.029 | 0 |
|  | Prescription drugs affecting thyroid function | -5.17 | -13.3 | -3.51 | 0.2 | 2.149 | 1 | 1.466 | 0 |
|  | Thyroid diseases | -5.59 | -6.4 | -3.52 | 0.4 | 2.111 | 1 | 1.453 | 0 |
| ln(Tg) | Crude/Full | -1.18 | Ref. | -1.07 | Ref. | 1.133 | 1 | 1.064 | 0 |
|  | Age | -1.38 | 16.9 | -1.07 | -0.2 | 1.879 | 1 | 1.371 | 0 |
|  | Sex | -1.21 | 3.0 | -1.10 | 3.1 | 1.482 | 1 | 1.217 | 0 |
|  | Race | -0.92 | -21.7 | -1.34 | 25.1 | 1.393 | 4 | 1.042 | 0 |
|  | Marry | -1.13 | -4.1 | -1.08 | 1.3 | 1.597 | 3 | 1.081 | 0 |
|  | PIR | -1.04 | -11.5 | -1.15 | 7.5 | 1.351 | 2 | 1.078 | 0 |
|  | Education | -1.15 | -2.5 | -1.10 | 2.3 | 1.394 | 2 | 1.086 | 0 |
|  | Alcohol user | -1.17 | -0.6 | -1.09 | 2.1 | 1.467 | 4 | 1.049 | 0 |
|  | CVD | -1.20 | 1.7 | -1.09 | 1.3 | 1.185 | 1 | 1.088 | 0 |
|  | Alt | -1.19 | 1.3 | -1.05 | -1.9 | 5.633 | 1 | 2.373 | 1 |
|  | Ast | -1.18 | 0.2 | -1.06 | -1.2 | 5.422 | 1 | 2.329 | 1 |
|  | Creatinine | -1.22 | 3.3 | -1.08 | 0.4 | 1.269 | 1 | 1.127 | 0 |
|  | Uric acid | -1.24 | 5.3 | -1.07 | 0.2 | 1.313 | 1 | 1.146 | 0 |
|  | Iodine urine | -1.16 | -1.7 | -1.08 | 1.2 | 1.064 | 1 | 1.032 | 0 |
|  | Prescription drugs affecting thyroid function | -1.48 | 25.8 | -1.03 | -3.6 | 2.184 | 1 | 1.478 | 0 |
|  | Thyroid diseases | -1.35 | 14.5 | -1.06 | -0.7 | 2.114 | 1 | 1.454 | 0 |
| ln(TgAb) | Crude/Full | 0.18 | Ref. | 0.30 | Ref. | 1.073 | 1 | 1.036 | 0 |
|  | Age | 0.59 | 235.7 | 0.21 | -29.4 | 1.882 | 1 | 1.372 | 0 |
|  | Sex | 0.13 | -23.9 | 0.29 | -3.7 | 1.473 | 1 | 1.214 | 0 |
|  | Race | 0.04 | -79.4 | 0.44 | 47.7 | 1.353 | 4 | 1.038 | 0 |
|  | Marry | 0.33 | 83.7 | 0.27 | -8.3 | 1.598 | 3 | 1.081 | 0 |
|  | PIR | 0.09 | -52.0 | 0.35 | 16.5 | 1.348 | 2 | 1.078 | 0 |
|  | Education | 0.12 | -33.1 | 0.32 | 6.9 | 1.396 | 2 | 1.087 | 0 |
|  | Alcohol user | 0.17 | -6.8 | 0.33 | 10.9 | 1.467 | 4 | 1.049 | 0 |
|  | CVD | 0.27 | 51.8 | 0.30 | 2.6 | 1.184 | 1 | 1.088 | 0 |
|  | Alt | 0.17 | -6.1 | 0.32 | 7.6 | 5.633 | 1 | 2.373 | 1 |
|  | Ast | 0.18 | -0.1 | 0.32 | 7.0 | 5.423 | 1 | 2.329 | 1 |
|  | Creatinine | 0.21 | 19.5 | 0.30 | 1.8 | 1.270 | 1 | 1.127 | 0 |
|  | Uric acid | 0.14 | -18.9 | 0.29 | -2.2 | 1.313 | 1 | 1.146 | 0 |
|  | Iodine urine | 0.19 | 6.5 | 0.30 | 0.6 | 1.060 | 1 | 1.029 | 0 |
|  | Prescription drugs affecting thyroid function | 0.37 | 110.1 | 0.29 | -2.0 | 2.138 | 1 | 1.462 | 0 |
|  | Thyroid diseases | 0.31 | 74.5 | 0.29 | -4.1 | 2.125 | 1 | 1.458 | 0 |
| ln(TPOAb) | Crude/Full | 0.16 | Ref. | 0.06 | Ref. | 1.084 | 1 | 1.041 | 0 |
|  | Age | 0.32 | 97.5 | 0.04 | -30.6 | 1.879 | 1 | 1.371 | 0 |
|  | Sex | 0.13 | -23.0 | 0.04 | -31.4 | 1.477 | 1 | 1.215 | 0 |
|  | Race | 0.05 | -70.1 | 0.17 | 199.8 | 1.358 | 4 | 1.039 | 0 |
|  | Marry | 0.22 | 34.6 | 0.05 | -17.6 | 1.595 | 3 | 1.081 | 0 |
|  | PIR | 0.04 | -75.0 | 0.13 | 132.6 | 1.354 | 2 | 1.079 | 0 |
|  | Education | 0.14 | -16.8 | 0.04 | -30.4 | 1.397 | 2 | 1.087 | 0 |
|  | Alcohol user | 0.15 | -9.2 | 0.06 | 11.9 | 1.465 | 4 | 1.049 | 0 |
|  | CVD | 0.14 | -13.2 | 0.08 | 39.7 | 1.186 | 1 | 1.089 | 0 |
|  | Alt | 0.15 | -5.9 | 0.06 | 7.4 | 5.632 | 1 | 2.373 | 1 |
|  | Ast | 0.16 | -1.1 | 0.05 | -5.9 | 5.422 | 1 | 2.328 | 1 |
|  | Creatinine | 0.15 | -6.2 | 0.06 | 4.7 | 1.269 | 1 | 1.127 | 0 |
|  | Uric acid | 0.10 | -35.8 | 0.04 | -19.7 | 1.313 | 1 | 1.146 | 0 |
|  | Iodine urine | 0.16 | -0.5 | 0.06 | 5.4 | 1.06 | 1 | 1.030 | 0 |
|  | Prescription drugs affecting thyroid function | 0.29 | 77.7 | 0.05 | -6.8 | 2.142 | 1 | 1.464 | 0 |
|  | Thyroid diseases | 0.25 | 54.2 | 0.05 | -12.0 | 2.125 | 1 | 1.458 | 0 |

**Notes:** Dependent variable: life’s essential 8 total score.

GVIF, generalized variance inflation factor; DF, degree of freedom.

GVIF^(1/(2*Df)) ≥ 2 indicates collinearity.
