# Supplementary material for: Association between thyroid hormone and cardiovascular health: A cross-sectional study
Source: PLoS One. 2025 Oct 24;20(10):e0329194. doi: 10.1371/journal.pone.0329194 (PMC12551862; doi:10.1371/journal.pone.0329194)
Supplement: S3 Table — (DOCX) [file pone.0329194.s023.docx]

**S3 Table. Association of covariates and life’s essential 8.**

| **Variables** | **Life’s essential 8** | |
| --- | --- | --- |
|  | ***β* (95%CI)** | ***p*-Value** |
| **Age** (years) | -0.23(-0.27, -0.19) | <0.001 |
| **Sex**, n (%) |  |  |
| Men | Reference |  |
| Women | 2.18(1.07, 3.29) | <0.001 |
| **Race****/ethnicity**, n (%) |  |  |
| Non-Hispanic White | Reference |  |
| Non-Hispanic Black | -5.01(-7.28, -2.73) | <0.001 |
| Mexican American | 1.74(-0.55, 4.04) | 0.133 |
| Other Hispanic | -0.44(-3.54, 2.66) | 0.775 |
| Other Race - Including Multi-Racial | 3.45(0.07, 6.83) | 0.046 |
| **Marital status**, n (%) |  |  |
| Married | Reference |  |
| Never married | 2.23(-0.13, 4.60) | 0.064 |
| Living with partner | -0.16(-2.63, 2.30) | 0.894 |
| Other: widowed, divorced, or separated individuals | -5.88(-7.21, -4.54) | <0.001 |
| **Education**, n (%) |  |  |
| Less Than 9th Grade | Reference |  |
| 9th Grade - High School Grade | 2.74(0.97, 4.51) | 0.003 |
| More Than High School Grade | 9.78(8.09, 11.46) | <0.001 |
| **Poverty income ratio**, n (%) |  |  |
| Low income (≤1.30) | Reference |  |
| Medium income (1.31-3.50) | 3.94(2.18, 5.69) | <0.001 |
| High income (>3.50) | 7.94(5.98, 9.90) | <0.001 |
| **Alcohol User**, n (%) |  |  |
| Never | Reference |  |
| Former | -4.77(-7.19, -2.35) | <0.001 |
| Mild | 2.14(0.31, 3.98) | 0.023 |
| Moderate | 2.51(-0.01, 5.03) | 0.051 |
| Heavy | -0.49(-3.03, 2.06) | 0.701 |
| **CVD**, n (%) |  |  |
| No | Reference |  |
| Yes | -10.49(-12.85, -8.12) | <0.001 |
| **Alt** (U/L) | -0.06(-0.11, -0.001) | 0.044 |
| **Ast** (U/L) | -0.03(-0.08, 0.02) | 0.260 |
| **Creatinine** (umol/L) | -5.60(-7.26, -3.94) | <0.001 |
| **Uric acid** (umol/L) | -2.68(-3.15, -2.22) | <0.001 |
| **Iodine, urine** (ug/L) | -0.0006(-0.0015, 0.0002) | 0.136 |
| **Prescription drugs affecting thyroid function**, n (%) |  |  |
| No | Reference |  |
| Yes | -3.89(-6.65, -1.12) | 0.007 |
| **Thyroid diseases**, n (%) |  |  |
| No | Reference |  |
| Yes | -2.54(-5.34, 0.25) | 0.074 |

***Abbreviation:*** CI, confidence interval; CVD, cardiovascular disease; Alt, alanine aminotransferase; Ast, aspartate aminotransferase.
