# Supplementary material for: Association between thyroid hormone and cardiovascular health: A cross-sectional study
Source: PLoS One. 2025 Oct 24;20(10):e0329194. doi: 10.1371/journal.pone.0329194 (PMC12551862; doi:10.1371/journal.pone.0329194)
Supplement: S4 Table — (DOCX) [file pone.0329194.s024.docx]

**S4 Table. Association between thyroid hormone and life’s essential 8 after excluding specific populations.**

| **Variables** | **LE8 total score** | | | |
| --- | --- | --- | --- | --- |
|  | **Cardiovascular disease-free cohort ^*^** | | **Thyroid disease-free cohort ^#^** | |
|  | ***β* (95%CI)** | ***p*-Value** | ***β* (95%CI)** | ***p*-Value** |
| **ln(TSH)** | -0.64(-1.49, 0.21) | 0.133 | -0.42(-1.20, 0.36) | 0.277 |
| **ln(FT3)** | -5.96(-11.93, 0.01) | 0.050 | -4.57(-8.57, -0.58) | 0.027 |
| **ln(FT4)** | 2.61(-0.29, 5.51) | 0.075 | 0.84(-1.45, 3.12) | 0.457 |
| **ln(TT3)** | -5.90(-9.09, -2.72) | <0.001 | -3.13(-5.17, -1.10) | 0.004 |
| **ln(TT4)** | -2.35(-5.16, 0.46) | 0.097 | -1.64(-3.76, 0.48) | 0.124 |
| **ln(Tg)** | -0.91(-1.74, -0.09) | 0.031 | -0.86(-1.54, -0.17) | 0.016 |
| **ln(TgAb)** | 0.21(-0.32, 0.74) | 0.417 | 0.38(-0.05, 0.80) | 0.079 |
| **ln(TPOAb)** | 0.07(-0.28, 0.41) | 0.682 | 0.13(-0.12, 0.37) | 0.304 |

***Abbreviation:*** CI, confidence interval; LE8, life’s essential 8; TSH, thyroid-stimulating hormone; FT3, free triiodothyronine; FT4, free thyroxine; TT3, total triiodothyronine; TT4, total thyroxine; Tg, thyroglobulin; TgAb, thyroglobulin antibodies; TPOAb, thyroid peroxidase antibody.

^*^ Model in cardiovascular disease-free cohort was adjusted for age, gender, race/ethnicity, urine iodine, education, marital status, PIR, creatinine, uric acid, Alt, Ast, alcohol user, prescription drugs affecting thyroid function, thyroid diseases. A total of 2669 participants were included.

^#^ Model in thyroid disease-free cohort was adjusted for age, gender, race/ethnicity, urine iodine, education, marital status, PIR, CVD, creatinine, uric acid, Alt, Ast, alcohol user, prescription drugs affecting thyroid function. A total of 2712 participants were included.
