# Supplementary material for: Association between thyroid hormone and cardiovascular health: A cross-sectional study
Source: PLoS One. 2025 Oct 24;20(10):e0329194. doi: 10.1371/journal.pone.0329194 (PMC12551862; doi:10.1371/journal.pone.0329194)
Supplement: S5 Table — (DOCX) [file pone.0329194.s025.docx]

**S5 Table. Association between thyroid hormone and life’s essential 8 under different missing data handling and weighting methods.**

| **Variables** | **LE8 total score** | | | | | |
| --- | --- | --- | --- | --- | --- | --- |
|  | **Under SAF weight and missing data deletion ^*^** | | **Under MEC weight and single imputation for missing data ^#^** | | **Under MEC weight and missing data deletion ^*#^** | |
|  | ***β* (95%CI)** | ***p*-Value** | ***β* (95%CI)** | ***p*-Value** | ***β* (95%CI)** | ***p*-Value** |
| **ln(TSH)** | -0.27(-0.84, 0.31) | 0.346 | -0.3(-0.64, 0.05) | 0.090 | -0.28(-0.65, 0.09) | 0.130 |
| **ln(FT3)** | -4.81(-9.25, -0.38) | 0.035 | -5.62(-8.70, -2.54) | <0.001 | -5.63(-8.83, -2.42) | 0.001 |
| **ln(FT4)** | 0.87(-0.90, 2.64) | 0.321 | 2.42(0.87, 3.97) | 0.004 | 1.98(0.34, 3.62) | 0.020 |
| **ln(TT3)** | -3.66(-5.81, -1.51) | 0.002 | -3.36(-4.89, -1.82) | <0.001 | -3.33(-4.93, -1.72) | <0.001 |
| **ln(TT4)** | -1.82(-3.73, 0.08) | 0.059 | -2.63(-4.16, -1.09) | 0.002 | -2.98(-4.50, -1.46) | <0.001 |
| **ln(Tg)** | -0.91(-1.43, -0.38) | 0.002 | -0.72(-0.99, -0.46) | <0.001 | -0.81(-1.08, -0.54) | <0.001 |
| **ln(TgAb)** | 0.35(0.01, 0.68) | 0.046 | 0.13(-0.10, 0.35) | 0.260 | 0.19(-0.04, 0.42) | 0.108 |
| **ln(TPOAb)** | 0.08(-0.18, 0.33) | 0.538 | 0.18（0.04, 0.33） | 0.015 | 0.17(0.01, 0.33) | 0.034 |

***Abbreviation:*** SAF weight, fasting subsample 2 year Mec weight; MEC, mobile examination center; MEC weight, full sample 2 year MEC exam weight; CI, confidence interval; LE8, life’s essential 8; TSH, thyroid-stimulating hormone; FT3, free triiodothyronine; FT4, free thyroxine; TT3, total triiodothyronine; TT4, total thyroxine; Tg, thyroglobulin; TgAb, thyroglobulin antibodies; TPOAb, thyroid peroxidase antibody.

Model was adjusted for age, gender, race/ethnicity, urine iodine, education, marital status, PIR, CVD, creatinine, uric acid, Alt, Ast, alcohol user, prescription drugs affecting thyroid function, thyroid diseases.

**^*^** A total of 2606 participants were included. **^#^** A total of 6566 participants were included. **^*#^** A total of 5657 participants were included.
