# Supplementary material for: Association between thyroid hormone and cardiovascular health: A cross-sectional study
Source: PLoS One. 2025 Oct 24;20(10):e0329194. doi: 10.1371/journal.pone.0329194 (PMC12551862; doi:10.1371/journal.pone.0329194)
Supplement: S1 Data — (DOCX) [file pone.0329194.s026.docx]

**Table. Database Access Links.**

| **Variable names in the manuscript** | **Names of data in NHANES** | **Cycle Year** | **Website for Data Download** |
| --- | --- | --- | --- |
| TSH | LBXTSH1 | 2007-2008 | https://wwwn.cdc.gov/nchs/nhanes/search/datapage.aspx?Component=Laboratory&Cycle=2007-2008 |
|  |  | 2009-2010 | https://wwwn.cdc.gov/Nchs/Nhanes/Search/DataPage.aspx?Component=Laboratory&Cycle=2009-2010 |
|  |  | 2011-2012 | https://wwwn.cdc.gov/Nchs/Nhanes/Search/DataPage.aspx?Component=Laboratory&Cycle=2011-2012 |
| FT3 | LBXT3F | 2007-2008 | https://wwwn.cdc.gov/nchs/nhanes/search/datapage.aspx?Component=Laboratory&Cycle=2007-2008 |
|  |  | 2009-2010 | https://wwwn.cdc.gov/Nchs/Nhanes/Search/DataPage.aspx?Component=Laboratory&Cycle=2009-2010 |
|  |  | 2011-2012 | https://wwwn.cdc.gov/Nchs/Nhanes/Search/DataPage.aspx?Component=Laboratory&Cycle=2011-2012 |
| FT4 | LBXT4F | 2007-2008 | https://wwwn.cdc.gov/nchs/nhanes/search/datapage.aspx?Component=Laboratory&Cycle=2007-2008 |
|  |  | 2009-2010 | https://wwwn.cdc.gov/Nchs/Nhanes/Search/DataPage.aspx?Component=Laboratory&Cycle=2009-2010 |
|  |  | 2011-2012 | https://wwwn.cdc.gov/Nchs/Nhanes/Search/DataPage.aspx?Component=Laboratory&Cycle=2011-2012 |
| TT3 | LBXTT3 | 2007-2008 | https://wwwn.cdc.gov/nchs/nhanes/search/datapage.aspx?Component=Laboratory&Cycle=2007-2008 |
|  |  | 2009-2010 | https://wwwn.cdc.gov/Nchs/Nhanes/Search/DataPage.aspx?Component=Laboratory&Cycle=2009-2010 |
|  |  | 2011-2012 | https://wwwn.cdc.gov/Nchs/Nhanes/Search/DataPage.aspx?Component=Laboratory&Cycle=2011-2012 |
| TT4 | LBXTT4 | 2007-2008 | https://wwwn.cdc.gov/nchs/nhanes/search/datapage.aspx?Component=Laboratory&Cycle=2007-2008 |
|  |  | 2009-2010 | https://wwwn.cdc.gov/Nchs/Nhanes/Search/DataPage.aspx?Component=Laboratory&Cycle=2009-2010 |
|  |  | 2011-2012 | https://wwwn.cdc.gov/Nchs/Nhanes/Search/DataPage.aspx?Component=Laboratory&Cycle=2011-2012 |
| Tg | LBXTGN | 2007-2008 | https://wwwn.cdc.gov/nchs/nhanes/search/datapage.aspx?Component=Laboratory&Cycle=2007-2008 |
|  |  | 2009-2010 | https://wwwn.cdc.gov/Nchs/Nhanes/Search/DataPage.aspx?Component=Laboratory&Cycle=2009-2010 |
|  |  | 2011-2012 | https://wwwn.cdc.gov/Nchs/Nhanes/Search/DataPage.aspx?Component=Laboratory&Cycle=2011-2012 |
| TgAb | LBXATG | 2007-2008 | https://wwwn.cdc.gov/nchs/nhanes/search/datapage.aspx?Component=Laboratory&Cycle=2007-2008 |
|  |  | 2009-2010 | https://wwwn.cdc.gov/Nchs/Nhanes/Search/DataPage.aspx?Component=Laboratory&Cycle=2009-2010 |
|  |  | 2011-2012 | https://wwwn.cdc.gov/Nchs/Nhanes/Search/DataPage.aspx?Component=Laboratory&Cycle=2011-2012 |
| TPOAb | LBXTPO | 2007-2008 | https://wwwn.cdc.gov/nchs/nhanes/search/datapage.aspx?Component=Laboratory&Cycle=2007-2008 |
|  |  | 2009-2010 | https://wwwn.cdc.gov/Nchs/Nhanes/Search/DataPage.aspx?Component=Laboratory&Cycle=2009-2010 |
|  |  | 2011-2012 | https://wwwn.cdc.gov/Nchs/Nhanes/Search/DataPage.aspx?Component=Laboratory&Cycle=2011-2012 |
| LE8 | Dietary Data | 2007-2008 | https://wwwn.cdc.gov/nchs/nhanes/search/datapage.aspx?Component=Dietary&Cycle=2007-2008 |
|  |  | 2009-2010 | https://wwwn.cdc.gov/nchs/nhanes/search/datapage.aspx?Component=Dietary&Cycle=2009-2010 |
|  |  | 2011-2012 | https://wwwn.cdc.gov/nchs/nhanes/search/datapage.aspx?Component=Dietary&Cycle=2011-2012 |
|  | Physical Activity | 2007-2008 | https://wwwn.cdc.gov/nchs/nhanes/search/datapage.aspx?Component=Questionnaire&Cycle=2007-2008 |
|  |  | 2009-2010 | https://wwwn.cdc.gov/nchs/nhanes/search/datapage.aspx?Component=Questionnaire&Cycle=2009-2010 |
|  |  | 2011-2012 | https://wwwn.cdc.gov/nchs/nhanes/search/datapage.aspx?Component=Questionnaire&Cycle=2011-2012 |
|  | Smoking | 2007-2008 | https://wwwn.cdc.gov/nchs/nhanes/search/datapage.aspx?Component=Questionnaire&Cycle=2007-2008 |
|  |  | 2009-2010 | https://wwwn.cdc.gov/nchs/nhanes/search/datapage.aspx?Component=Questionnaire&Cycle=2009-2010 |
|  |  | 2011-2012 | https://wwwn.cdc.gov/nchs/nhanes/search/datapage.aspx?Component=Questionnaire&Cycle=2011-2012 |
|  | Sleep | 2007-2008 | https://wwwn.cdc.gov/nchs/nhanes/search/datapage.aspx?Component=Questionnaire&Cycle=2007-2008 |
|  |  | 2009-2010 | https://wwwn.cdc.gov/nchs/nhanes/search/datapage.aspx?Component=Questionnaire&Cycle=2009-2010 |
|  |  | 2011-2012 | https://wwwn.cdc.gov/nchs/nhanes/search/datapage.aspx?Component=Questionnaire&Cycle=2011-2012 |
|  | Body Mass Index | 2007-2008 | https://wwwn.cdc.gov/Nchs/Nhanes/Search/DataPage.aspx?Component=Examination&Cycle=2007-2008 |
|  |  | 2009-2010 | https://wwwn.cdc.gov/Nchs/Nhanes/Search/DataPage.aspx?Component=Examination&Cycle=2009-2010 |
|  |  | 2011-2012 | https://wwwn.cdc.gov/Nchs/Nhanes/Search/DataPage.aspx?Component=Examination&Cycle=2011-2012 |
|  | Blood Pressure | 2007-2008 | https://wwwn.cdc.gov/Nchs/Nhanes/Search/DataPage.aspx?Component=Examination&Cycle=2007-2008 |
|  |  | 2009-2010 | https://wwwn.cdc.gov/Nchs/Nhanes/Search/DataPage.aspx?Component=Examination&Cycle=2009-2010 |
|  |  | 2011-2012 | https://wwwn.cdc.gov/Nchs/Nhanes/Search/DataPage.aspx?Component=Examination&Cycle=2011-2012 |
|  | Cholesterol | 2007-2008 | https://wwwn.cdc.gov/nchs/nhanes/search/datapage.aspx?Component=Laboratory&Cycle=2007-2008 |
|  |  | 2009-2010 | https://wwwn.cdc.gov/Nchs/Nhanes/Search/DataPage.aspx?Component=Laboratory&Cycle=2009-2010 |
|  |  | 2011-2012 | https://wwwn.cdc.gov/Nchs/Nhanes/Search/DataPage.aspx?Component=Laboratory&Cycle=2011-2012 |
|  | Blood Glucose | 2007-2008 | https://wwwn.cdc.gov/nchs/nhanes/search/datapage.aspx?Component=Laboratory&Cycle=2007-2008 |
|  |  | 2009-2010 | https://wwwn.cdc.gov/Nchs/Nhanes/Search/DataPage.aspx?Component=Laboratory&Cycle=2009-2010 |
|  |  | 2011-2012 | https://wwwn.cdc.gov/Nchs/Nhanes/Search/DataPage.aspx?Component=Laboratory&Cycle=2011-2012 |
| Age | RIDAGEYR | 2007-2008 | https://wwwn.cdc.gov/nchs/nhanes/search/datapage.aspx?Component=Demographics&Cycle=2007-2008 |
|  |  | 2009-2010 | https://wwwn.cdc.gov/nchs/nhanes/search/datapage.aspx?Component=Demographics&Cycle=2009-2010 |
|  |  | 2011-2012 | https://wwwn.cdc.gov/nchs/nhanes/search/datapage.aspx?Component=Demographics&Cycle=2011-2012 |
| Sex | RIAGENDR | 2007-2008 | https://wwwn.cdc.gov/nchs/nhanes/search/datapage.aspx?Component=Demographics&Cycle=2007-2008 |
|  |  | 2009-2010 | https://wwwn.cdc.gov/nchs/nhanes/search/datapage.aspx?Component=Demographics&Cycle=2009-2010 |
|  |  | 2011-2012 | https://wwwn.cdc.gov/nchs/nhanes/search/datapage.aspx?Component=Demographics&Cycle=2011-2012 |
| Race/ethnicity | RIDRETH1 | 2007-2008 | https://wwwn.cdc.gov/nchs/nhanes/search/datapage.aspx?Component=Demographics&Cycle=2007-2008 |
|  |  | 2009-2010 | https://wwwn.cdc.gov/nchs/nhanes/search/datapage.aspx?Component=Demographics&Cycle=2009-2010 |
|  |  | 2011-2012 | https://wwwn.cdc.gov/nchs/nhanes/search/datapage.aspx?Component=Demographics&Cycle=2011-2012 |
| Education | DMDEDUC2 | 2007-2008 | https://wwwn.cdc.gov/nchs/nhanes/search/datapage.aspx?Component=Demographics&Cycle=2007-2008 |
|  |  | 2009-2010 | https://wwwn.cdc.gov/nchs/nhanes/search/datapage.aspx?Component=Demographics&Cycle=2009-2010 |
|  |  | 2011-2012 | https://wwwn.cdc.gov/nchs/nhanes/search/datapage.aspx?Component=Demographics&Cycle=2011-2012 |
| Marital status | DMDMARTL | 2007-2008 | https://wwwn.cdc.gov/nchs/nhanes/search/datapage.aspx?Component=Demographics&Cycle=2007-2008 |
|  |  | 2009-2010 | https://wwwn.cdc.gov/nchs/nhanes/search/datapage.aspx?Component=Demographics&Cycle=2009-2010 |
|  |  | 2011-2012 | https://wwwn.cdc.gov/nchs/nhanes/search/datapage.aspx?Component=Demographics&Cycle=2011-2012 |
| Poverty income ratio | INDFMPIR | 2007-2008 | https://wwwn.cdc.gov/nchs/nhanes/search/datapage.aspx?Component=Demographics&Cycle=2007-2008 |
|  |  | 2009-2010 | https://wwwn.cdc.gov/nchs/nhanes/search/datapage.aspx?Component=Demographics&Cycle=2009-2010 |
|  |  | 2011-2012 | https://wwwn.cdc.gov/nchs/nhanes/search/datapage.aspx?Component=Demographics&Cycle=2011-2012 |
| Alcohol user | Alcohol Use | 2007-2008 | https://wwwn.cdc.gov/nchs/nhanes/search/datapage.aspx?Component=Questionnaire&Cycle=2007-2008 |
|  |  | 2009-2010 | https://wwwn.cdc.gov/nchs/nhanes/search/datapage.aspx?Component=Questionnaire&Cycle=2009-2010 |
|  |  | 2011-2012 | https://wwwn.cdc.gov/nchs/nhanes/search/datapage.aspx?Component=Questionnaire&Cycle=2011-2012 |
| Uric acid | LBXSUA | 2007-2008 | https://wwwn.cdc.gov/nchs/nhanes/search/datapage.aspx?Component=Laboratory&Cycle=2007-2008 |
|  |  | 2009-2010 | https://wwwn.cdc.gov/Nchs/Nhanes/Search/DataPage.aspx?Component=Laboratory&Cycle=2009-2010 |
|  |  | 2011-2012 | https://wwwn.cdc.gov/Nchs/Nhanes/Search/DataPage.aspx?Component=Laboratory&Cycle=2011-2012 |
| Creatinine | LBXSCR | 2007-2008 | https://wwwn.cdc.gov/nchs/nhanes/search/datapage.aspx?Component=Laboratory&Cycle=2007-2008 |
|  |  | 2009-2010 | https://wwwn.cdc.gov/Nchs/Nhanes/Search/DataPage.aspx?Component=Laboratory&Cycle=2009-2010 |
|  |  | 2011-2012 | https://wwwn.cdc.gov/Nchs/Nhanes/Search/DataPage.aspx?Component=Laboratory&Cycle=2011-2012 |
| CVD | Medical Conditions | 2007-2008 | https://wwwn.cdc.gov/nchs/nhanes/search/datapage.aspx?Component=Questionnaire&Cycle=2007-2008 |
|  |  | 2009-2010 | https://wwwn.cdc.gov/nchs/nhanes/search/datapage.aspx?Component=Questionnaire&Cycle=2009-2010 |
|  |  | 2011-2012 | https://wwwn.cdc.gov/nchs/nhanes/search/datapage.aspx?Component=Questionnaire&Cycle=2011-2012 |
| Alt | LBXSATSI | 2007-2008 | https://wwwn.cdc.gov/nchs/nhanes/search/datapage.aspx?Component=Laboratory&Cycle=2007-2008 |
|  |  | 2009-2010 | https://wwwn.cdc.gov/Nchs/Nhanes/Search/DataPage.aspx?Component=Laboratory&Cycle=2009-2010 |
|  |  | 2011-2012 | https://wwwn.cdc.gov/Nchs/Nhanes/Search/DataPage.aspx?Component=Laboratory&Cycle=2011-2012 |
| Ast | [LBXSASSI](https://wwwn.cdc.gov/Nchs/Data/Nhanes/Public/2011/DataFiles/BIOPRO_G.htm#LBXSASSI) | 2007-2008 | https://wwwn.cdc.gov/nchs/nhanes/search/datapage.aspx?Component=Laboratory&Cycle=2007-2008 |
|  |  | 2009-2010 | https://wwwn.cdc.gov/Nchs/Nhanes/Search/DataPage.aspx?Component=Laboratory&Cycle=2009-2010 |
|  |  | 2011-2012 | https://wwwn.cdc.gov/Nchs/Nhanes/Search/DataPage.aspx?Component=Laboratory&Cycle=2011-2012 |
| Iodine, urine | Iodine - Urine | 2007-2008 | https://wwwn.cdc.gov/nchs/nhanes/search/datapage.aspx?Component=Laboratory&Cycle=2007-2008 |
|  |  | 2009-2010 | https://wwwn.cdc.gov/Nchs/Nhanes/Search/DataPage.aspx?Component=Laboratory&Cycle=2009-2010 |
|  |  | 2011-2012 | https://wwwn.cdc.gov/Nchs/Nhanes/Search/DataPage.aspx?Component=Laboratory&Cycle=2011-2012 |
| Thyroid diseases | Medical Conditions | 2007-2008 | https://wwwn.cdc.gov/nchs/nhanes/search/datapage.aspx?Component=Questionnaire&Cycle=2007-2008 |
|  |  | 2009-2010 | https://wwwn.cdc.gov/nchs/nhanes/search/datapage.aspx?Component=Questionnaire&Cycle=2009-2010 |
|  |  | 2011-2012 | https://wwwn.cdc.gov/nchs/nhanes/search/datapage.aspx?Component=Questionnaire&Cycle=2011-2012 |
| Prescription drugs affecting thyroid function | Prescription Medications | 2013-2014 | [https://wwwn.cdc.gov/Nchs/Data/Nhanes/Public/2013/DataFiles/RXQ_RX_H.htm#Appendix:_ICD-10-CM_codes_assigned_to_reasons_for_use_reported_by_participants](https://wwwn.cdc.gov/Nchs/Data/Nhanes/Public/2013/DataFiles/RXQ_RX_H.htm#Appendix:_ICD-10-CM_codes_assigned_to_reasons_for_use_reported_by_participants.) |
|  | Prescription Medications - Drug Information | 1988-2020 | https://wwwn.cdc.gov/Nchs/Data/Nhanes/Public/1988/DataFiles/RXQ_DRUG.htm |

***Abbreviation:*** TSH, thyroid-stimulating hormone; FT3, free triiodothyronine; FT4, free thyroxine; TT3, total triiodothyronine; TT4, total thyroxine; Tg, thyroglobulin; TgAb, thyroglobulin antibodies; TPOAb, thyroid peroxidase antibody; LE8, life’s essential 8; Alt, alanine aminotransferase; Ast, aspartate aminotransferase; CVD, cardiovascular disease.
